# HDAC5 modulates PD-L1 expression and cancer immunity via p65 deacetylation in pancreatic cancer

**DOI:** 10.7150/thno.69444

**Published:** 2022-01-31

**Authors:** Yingke Zhou, Xin Jin, Haixin Yu, Gengdu Qin, Penglin Pan, Jingyuan Zhao, Taoyu Chen, Xueyi Liang, Yan Sun, Bo Wang, Dianyun Ren, Shikai Zhu, Heshui Wu

**Affiliations:** 1 Department of Pancreatic Surgery, Union Hospital, Tongji Medical College, Huazhong University of Science and Technology, Wuhan, 430022 China.; 2 Sino-German Laboratory of Personalized Medicine for Pancreatic Cancer, Union Hospital, Tongji Medical College, Huazhong University of Science and Technology, Wuhan, 430022 China.; 3 Department of Urology, The Second Xiangya Hospital, Central South University, Changsha, Hunan, 410011, China.; 4 Uro-Oncology Institute of Central South University, Changsha, Hunan, 410011, China.; 5 Cancer Center, Union Hospital, Tongji Medical College, Huazhong University of Science and Technology, Wuhan, 430022 China.; 6 Clinical Immunology Translational Medicine Key Laboratory of Sichuan Province, Organ Transplant Center, Sichuan Provincial People's Hospital, University of Electronic Science and Technology of China, Chengdu, 611731 China.

**Keywords:** Pancreatic cancer, Immunotherapy, PD-L1, HDAC5, NF-κB

## Abstract

**Rationale:** Pancreatic ductal adenocarcinoma (PDAC) is a lethal disease with a dismal 5-year survival less than 10%. Most patients with PDAC exhibit poor response to single-agent immunotherapy. Multimodal therapies targeting mechanisms of resistance to immunotherapy are urgently needed. We found that the class IIa histone deacetylase (HDAC) member, HDAC5 is downregulated in multiple solid tumors and its level were associated with favorable prognosis in PDAC patients. Upregulated genes in patients harboring HDAC5 deletions were enriched in adaptive immune responses and lymphocyte-mediated immunity in The Cancer Genome Atlas (TCGA) pancreatic cancer dataset.

**Methods:** Tissue microarray of pancreatic cancer were used to analysis the correlation between HDAC5 and PD-L1. RNA-seq, transcription factor motif analysis, drug screening and molecular biology assays were performed to identify the mechanism of HDAC5's repression on PD-L1. Allografts of pancreatic cancer in mouse were applied to test the efficiency of HDAC5 inhibition and anti-PD1 co-treatment.

**Results:** HDAC5 regulated PD-L1 expression by directly interacting with NF-κB p65; this interaction was suppressed by p65 phosphorylation at serine-311. Additionally, HDAC5 diminished p65 acetylation at lysine-310, which is essential for the transcriptional activity of p65. Importantly, we demonstrated that HDAC5 silencing or inhibition sensitized PDAC tumors to immune checkpoint blockade (ICB) therapy in syngeneic mouse model and KPC mouse derived PDAC model.

**Conclusion:** Our findings revealed a previously unknown role of HDAC5 in regulating the NF-κB signaling pathway and antitumor immune responses. These findings provide a strong rationale for augment the antitumor effects of ICB in immunotherapy-resistant PDAC by inhibiting HDAC5.

## Introduction

Pancreatic ductal adenocarcinoma (PDAC) is considered as one of the most immunotherapy-resistant cancer types 1. Single-agent immune checkpoint blockade (ICB) therapies in PDAC have proved to be ineffective. Therefore, multimodal therapies targeting mechanisms of resistance to immunotherapy are urgently needed 2. Recent studies have shown that activating *KRAS* mutations may induce an immunosuppressive tumor microenvironment (TME) by modulating major histocompatibility complex class I (MHC-I) via autophagocytosis 3, 4, as well as by regulating the level of PD-L1 5. Tumor mutational burden 6; deregulated expression of PD-L1, PD-1, and CD8; and T cell clonality have also been linked to clinical response to anti- PD-L1/ PD-1therapies 7. Thus, further studies are critical to unveil the specific mechanisms underlying the immune checkpoint molecules to improve PDAC response to immunotherapy.

Dysregulation of histone deacetylases (HDACs) are highly implicated in cancer 8-10. So far, several HDAC inhibitors have already been implemented as anti-neoplastic agents in the clinic or ongoing clinical trials 8-10. Interestingly, pan-HDAC inhibitors, such as Vorinostat and Trichostatin A (TSA), have been shown to diminish immune responses 11, 12; however, the underlying mechanisms remain elusive. Notably, HDAC5 has been demonstrated to reduce the immune response and *de novo* increasing of T regulatory (Treg) cells 13, implying the importance of HDAC5 in antitumor immune responses. HDAC5 is an important member of class IIa HDACs (in addition to HDAC4, 7, and 9), which have a higher molecular weight (120-135 kDa) than other HDACs and distinct catalytic mechanisms 14. The role of HDAC5 in cancer remains controversial; HDAC5 may act as a tumor suppressor or oncogene in a tissue-specific or tumor subtype-dependent manner 15-17.

NF-κB is a transcription factor implicated in inflammation, immunity, and cancer 18. NF-κB signaling is activated by oxidative stress, DNA damage, necrotic cell products, bacterial infections, and pro-inflammatory cytokines 19. In PDAC or other cancers, NF-κB is constitutively active due to increased levels of IκB kinase (IKK)-activating cytokines, such as IL-1 and tumor necrosis factor (TNF) 20, 21. NF-κB is critical in regulating both adaptive and innate immune responses 19. Furthermore, NF-κB regulates the level of *CD274* (encoding PD-L1) in various cancer types 22, 23. A CRISPR/Cas9-based screening identified NF-κB as a crucial mediator of immune evasion of cancer cells 24, 25. In this study, we identified HDAC5 was a key regulator of PD-L1 through mediating the deacetylation of NF-κB p65 in pancreatic cancer.

## Materials and Methods

### Cell culture and transfection

All the cell lines in this study (293T, PANC-1, Panc 02, Mia PaCa-2) were cultured in Dulbecco's Modified Eagle Medium (DMEM) (Invitrogen, USA) supplemented with 10% fetal bovine serum (FBS) (Hyclone, USA). Cells were cultured at 37 °C with 5% CO2. Mycoplasma contamination was regularly examined using Lookout Mycoplasma PCR Detection Kit (Sigma-Aldrich). Cell lines were periodically authenticated via STR profiling (Procell, China). 2-5 μL of Lipofectamine 2000 were used for the transfection of 1 μg plasmid. Lipofectamine 2000 and plasmid were dilute with Opti-MEM separately, then were mixed together and incubated at room temperature for 5 min. The DNA-lipid complex was added to cells after incubation.

### RNA interference

Lentivirus-based small hairpin RNAs (shRNA) were from Sigma-Aldrich. psPAX2 and pMD2.G combined with specific shRNA were co-transfected into 293T cells. Replace the culture medium with fresh DMEM with 10% FBS 24 h after transfection. 48 h later, the virus containing medium was collected and added to cancer cells with polybrene (12 μg/mL). Puromycin selection was performed at concentration ranges between 3-5 μg/mL 48 h after infection. The sequence information of shRNA is provided in table S1.

### Quantitative RT-PCR

TRIzol (Thermo Fisher Scientific) were used to extract RNA from cancer cells. 2 μg RNA were used to synthesis cDNA via following the instruction of PrimeScript™ RT reagent Kit (Code No. RR037A, Takara Bio Inc., Shigo, Japan). The quantitative PCR was performed by using TB Green™ Fast qPCR Mix PCR kit (Code No. RR430A, Takara Bio Inc., Shigo, Japan). The '2-^ΔΔ^C(T)' method was used to calculate the fold change by normalizing to Actin. Primers with 90%-110% amplification efficiency were used to perform quantification analysis. The sequence of primers used for RT-qPCR is provided in Table S1.

### Co-immunoprecipitation (co-IP) and Western Blot

Cells were collected and lysed by 1 mL lysis buffer (Beyotime, China, containing 20 mM Tris (pH 7.5), 150 mM NaCl, 1% Triton X-100, sodium pyrophosphate, β-glycerophosphate, EDTA, Na3VO4 and leupeptin) with 1 mM PMSF on ice for 0.5 h. Then, cell lysate was centrifuged at 13200 rpm at 4 °C for 15 min. Then, protein A/G agarose beads (Thermo Fisher Scientific, USA) and primary antibody were added to the supernatant and incubated at 4 °C overnight. The beads were washed with lysis buffer on ice for six times, and the beads were then subjected to western blot analysis.

The protein concentration was quantified by a protein assay kit (Pierce Biotechnology, USA). Samples containing protein in same amount were subjected to SDS-PAGE analysis and transferred to PVDF membranes. 1 × TBST with 5% non-fat milk powder were used to block the membrane for 2 h at room temperature. Then primary antibody was used to incubate with the membrane at 4 °C overnight. 1 × TBST was applied to wash the membrane for three times. Finally, horseradish peroxidase-conjugated secondary antibodies were used to incubate the membrane for 2 h at room temperature. The protein bands were visualized by ECL detection reagents. Working dilution of antibodies is provided in Table S2.

### Chromatin immunoprecipitation (ChIP) and ChIP-qPCR

Chromatin Extraction Kit (Abcam, USA) and ChIP Kit Magnetic - One Step (Abcam, USA) were used to perform ChIP. The purified DNA was analyzed by the same methods as the cDNA in RT-qPCR. The primers for ChIP-qPCR are provided in Supplementary Table S1.

### Flow cytometry analysis of the mouse allograft samples

Tumors were divided into small pieces and added with 2 mg/ml collagenase (Sigma, USA) in DMEM for 1 h at 37 °C. A 70 μm nylon strainer was used to filter the digested cells. The cells were then suspended in PBS with 2% BSA and co-stained with the indicated antibodies (The detailed antibody information is provided in supplementary data). The cells were incubated in antibodies for 15 min, washed with PBS and then sent to the analysis by flow cytometry.

### Tissue microarray and immunohistochemistry (IHC)

The tissue microarray (TMA) slides origin is Outdo Biobank (Shanghai, China) (HPan-Ade060CD-01). The TMA slides were immuno-stained with PD-L1 (1:1000) and HDAC5 (1:5000) specific antibodies. Score in blinded fashion were used to assess the staining intensity: 1 = weak staining at 100× magnification but little or no staining at 40× magnification; 2 = medium staining at 40× magnification; 3 = strong staining at 40× magnification. Two independent pathologists performed the assessment. The staining intensity was calculated by the function: SI = (positive cells% * the staining intensity).

### Generation of the allograft pancreatic cancer mouse model for anti-PD1 antibody therapy

6-week-old C57BL/6 mice were from Charles River Laboratories (Wuhan, China). The study was approved by the Ethics Committee of Tongji Medical College, Huazhong University of Science and Technology. Panc02 cells (5 × 10^6^ in 100 µL 1 × PBS) were injected s.c. into the right flank of mice. The volume of the allograft was calculated by the formula (L × W^2^ × 0.5). After the allografts reached a size of 50 mm^3^, mice carrying same types of tumors were divided randomly (simple randomization) into different groups and treated with anti-PD-1 (BioXcell, Clone RMP1-14) or IgG (BioXcell, Clone 2A3) with or without the co-treatment of LMK235 (MedChemExpress, HY-18998). Mice were euthanized at day 24 (mice meet the standard for euthanize before the end-day because of unexpected reasons like ulceration or infection were excluded from the analysis). Tumors were collected for flow cytometry analysis. KPC derived PDAC cells were cultured as described previously 26. 10^5^ KPC derived PDAC cells were injected orthotopically into C57BL/6 mice. Mouse was euthanized when it meets the end-point standard required by ethics committee. And the tumor was collected for immune-fluorescence and FACS.

### Glutathione S-transferase (GST) pull down assay

Cells were lysed with IP buffer on ice for more than 30 min. Glutathione-Sepharose beads (GE Healthcare Lifesciences) were used to immobilize GST fusion proteins. After immobilization, the beads were incubated with cell lysates at 4 °C for 8 h. Binding buffer were used to wash the beads for at least six times. Then the bound proteins were analyzed by western blotting assay.

### RNA sequence

Total RNA was prepared by Trizol (Invitrogen, Cata No. 15596026). RNA sequence was performed by Novogene (Beijing, China) on the illumine sequence platform. RNA degradation and contamination were monitored on 1% agarose gels and RNA purity was checked using the NanoPhotometer spectrophotometer (IMPLEN, CA, USA), RNA integrity was assessed using the RNA Nano 6000 Assay Kit of the Bioanalyzer 2100 system (Agilent Technologies, CA, USA). The clustering of the index-coded samples was performed on a cBot Cluster Generation System using TruSeq PE Cluster Kit v3-cBot-HS (Illumia) according to the manufacturer's instructions. The library preparations were sequenced on an Illumina Novaseq platform and 150 bp paired-end reads were generated.

### Statistical analysis

The differential expressed genes (DEGs) in TCGA pancreatic cancer dataset was obtained by DESeq2 (http://bioconductor.org/packages/release/bioc/html/DESeq2.html). Genes with *P* < 0.05 were identified as differentially expressed between two groups of patients. Transcriptional factor binding motif analysis was performed on Alibaba2 (http://gene-regulation.com/pub/programs/alibaba2/). The variances of the groups being statistically compared are similar. And the group size is the minimum that is sufficient to reliably define a specific functional parameter taking into account the biological variability. Statistical analyses without annotation were performed with one-sided or two-sided paired Student's t-test for single comparison. Multiple comparisons were analyzed with One-way ANOVA with a post hoc test. We consider the result is statistically significant when a *P* value < 0.05.

Sequence of primers, shRNAs, SiRNAs, information of antibodies, chemicals and recombinant DNA are provided in supplementary materials.

## Results

### HDAC5 negatively regulates PD-L1 expression in pancreatic cancer

**Figure 1:**
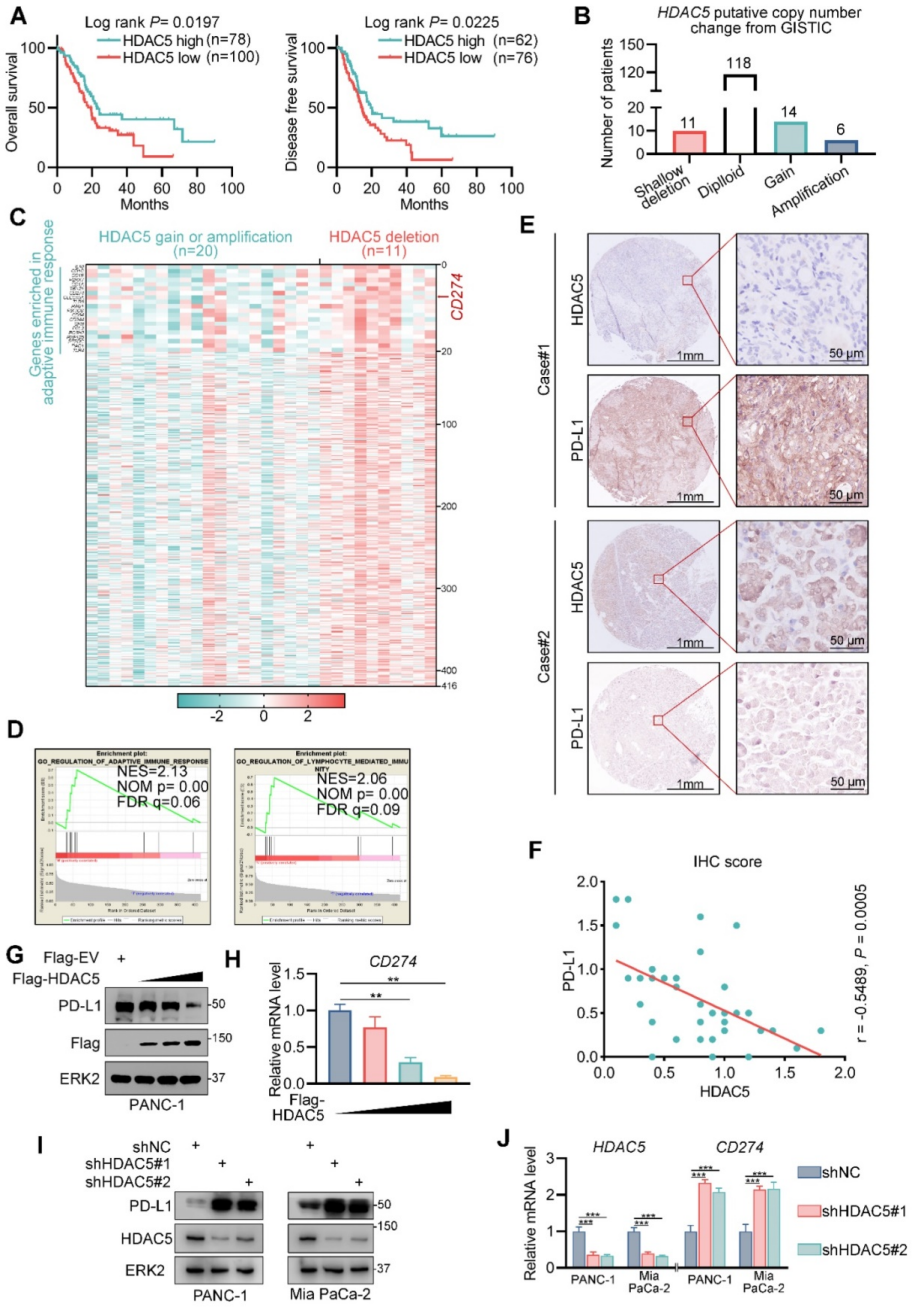
** HDAC5 negatively regulates PD-L1 in pancreatic cancer. (A)** Kaplan-Meier plots showing the overall survival and disease-free survival between HDAC5 high and low patients in TCGA PAAD dataset. **(B)** Histogram showing the distribution of *HDAC5* putative copy number change from GSITIC in TCGA pancreatic cancer dataset. **(C)** Heatmap showing the upregulated genes in HDAC5 shallow deletion patients compared to HDAC5 gain or amplification patients (log_2_[fold change] > 0.1, *P* < 0.05). **(D)** GSEA output showing the most enriched pathways based on the upregulated genes in Fig. 1c. **(E)** Images of IHC analysis for HDAC5 and PD-L1 in PDAC patient samples (n = 35). **(F)** Correlation analysis of IHC staining index of HDAC5 and PD-L1 in PDAC patient specimens (n = 35). Spearman correlation coefficient and *P* value are shown. **(G-H)** PANC-1 cells were transfected with indicated plasmids in a gradient dose (0, 1, 3, 5 µg). 48 h after transfection, cells were harvested for western blot analysis (G) and RT-qPCR, data are shown as mean ± SD (n = 3, ** *P* < 0.01) (H). **(I-J)** PANC-1 and Mia PaCa-2 cells were infected with lenti-virus expressing indicated shRNA for 48 h. After 48 h puromycin selection, cells were harvested for western blot analysis (I) and RT-qPCR, data are shown as mean ± SD (n = 3, * *P* < 0.05, ** *P* < 0.01) (J).

To elucidate the role of class IIa HDAC members in pancreatic cancer, we explored their association with patient survival in the TCGA pancreatic cancer dataset. We found that HDAC5 levels were associated with favorable overall survival and disease-free survival (Figure 1A). We also analyzed the differentially expressed genes between tumors with HDAC5 shallow deletion and those with HDAC5 gain or amplification (Figure 1B). We identified 416 upregulated genes in tumors with HDAC5 deletion (Figure 1C); *CD274* was among these upregulated genes (Figure 1C). Gene set enrichment analysis demonstrated that these genes were significantly enriched in gene sets related to adaptive immune responses and lymphocyte-mediated immunity (Figure 1C-D).

Anti-PD-1/ PD-L1 agents are already used for multiple solid malignancies; however, most pancreatic cancer patients do not benefit from these therapies. Hence, we next assessed the role of HDAC5 in PD-L1 expression. To this end, we performed immunohistochemistry on a tissue microarray of pancreatic cancer specimens (Figure 1E). We showed that the expression of PD-L1 were negatively correlated with those of HDAC5 (Spearman correlation coefficient: -0.4497, *P* = 0.0028) (Figure 1F). Furthermore, ectopic expression of HDAC5 in PANC-1 cells reduced PD-L1 mRNA and protein levels (Figure 1G-H; Figure S1A). And HDAC5 knockdown in 2 different pancreatic cancer cell lines (Mia PaCa-2 and PANC-1) using 2 independent shRNAs increased PD-L1 mRNA and protein levels (Figure 1I-J; Figure S1B). These data suggest that HDAC5 represses PD-L1 expression at the transcriptional level.

### HDAC5 regulates PD-L1 expression in an NF-κB-dependent manner

**Figure 2:**
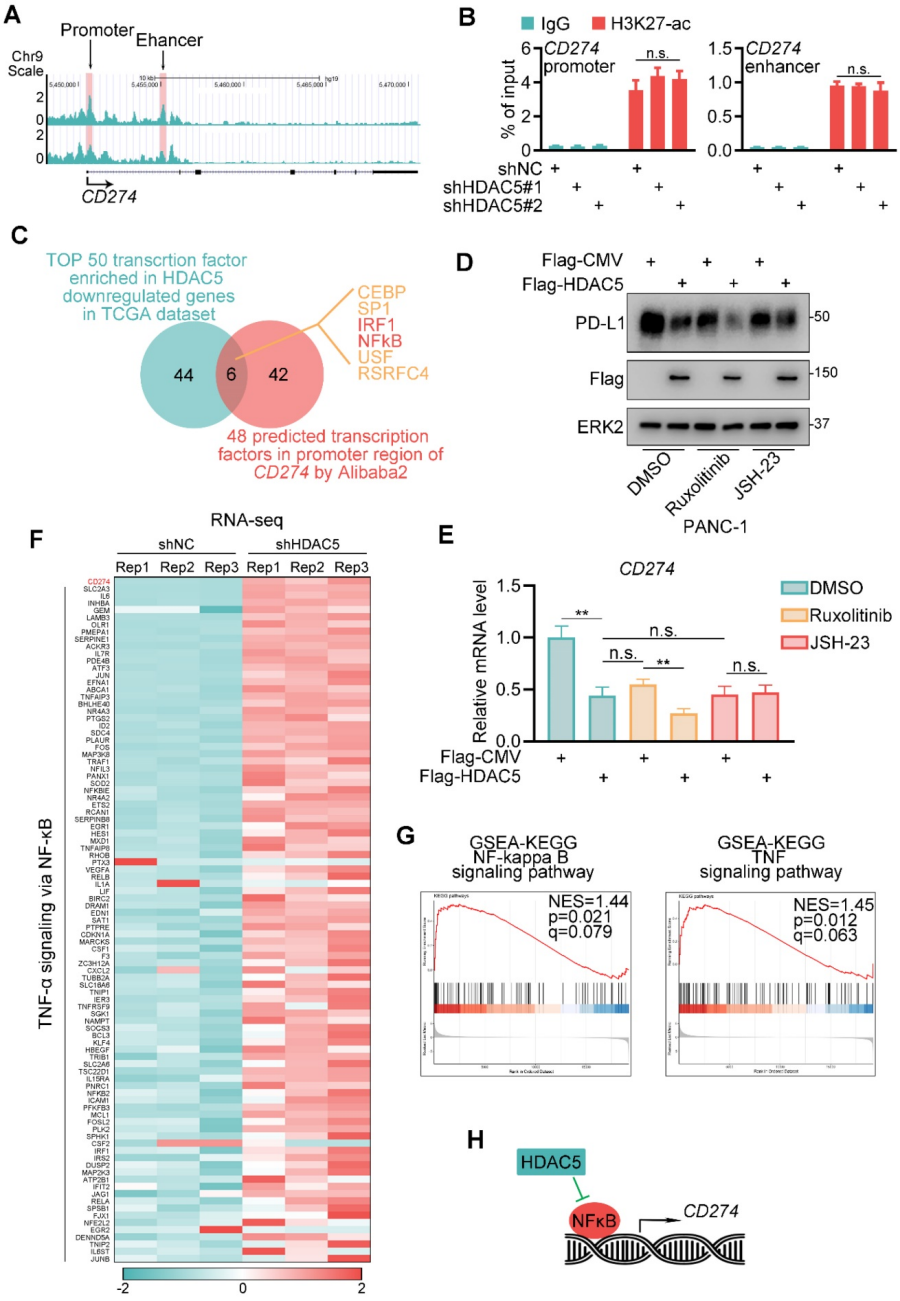
** HDAC5 modulates NF-κB dependent expression of PD-L1. (A)** UCSC Genome Browser screen shot of H3K27-ac ChIP-seq tracks at the gene loci of *CD274.* **(B)** PANC-1 cells were infected with lenti-virus expressing indicated shRNAs for 48 h. After 48 h puromycin selection, cells were harvested for ChIP-qPCR analysis to detect the H3K27-ac enrichment at promoter or enhancer region of *CD274*. Data are shown as mean ± SD (n = 3, n.s. not significant). **(C)** Venn diagram depicting the predicted HDAC5 regulated transcription factor of *CD274*. **(D-E)** PANC-1 cells were transfected with indicated plasmid. 24 h after transfection, cells were treated with indicated drugs for 24 h (2.5 µM Ruxolitinib, 10 µM JSH-23). Then cells were harvested for western blot analysis (D) and RT-qPCR. Data are shown as mean ± SD (n = 3, n.s. not significant, ** *P* < 0.01) (E). **(F)** Heatmap showing the expression of CD274 and a subset of genes involved in TNF-α-NF-κB pathway. **(G)** Gene set enrichment analysis (GSEA) of the indicated pathways. **(H)** Model depicting the mechanism that HDAC5 regulates PD-L1 expression in an NF-κB dependent manner.

We next assessed whether HDAC5 regulates histone acetylation in the gene locus of *CD274.* Surprisingly, HDAC5 silencing did not affect H3K27ac levels in the promoter or enhancer region of *CD274* (Figure 2A-B). Then we performed a transcription factor binding motif analysis of the *CD274* promoter region*.* We identified 6 putative transcription factor motifs, the transcriptional activity of which may be repressed by HDAC5 (Figure 2C). Some of these sites were predicted to be recognized by well-characterized transcriptional regulators of PD-L1, such as IRF1 and NF-κB 27, 28. Ectopic expression of HDAC5 had minimal effect on PD-L1 expression levels in the presence of the NF-κB inhibitor JSH-23 but not in the presence of the JAK inhibitor ruxolitinib (Figure 2D-E; Figure S2A-D). Consistently, HDAC5 knockdown failed to upregulate PD-L1 in the presence of JSH-23 (Figure S2E-F).

To further verify HDAC5's function on NF-κB signaling and PD-L1 expression, we performed RNA-seq analysis in control and HDAC5 knock-down PANC-1 cells. Consistently, CD274 is differentially upregulated by the knock-down of HDAC5 (Figure S2G). And gene set enrichment analysis (GSEA) indicated that HDAC5 knock-down resulted in a significant activation of NF-κB signaling and TNF signaling (Figure 2F-G). Taken together, our data suggest that the ability of HDAC5 to regulate PD-L1 expression is dependent on the NF-κB pathway (Figure 2H).

### HDAC5 interacts with p65 in pancreatic cancer

**Figure 3:**
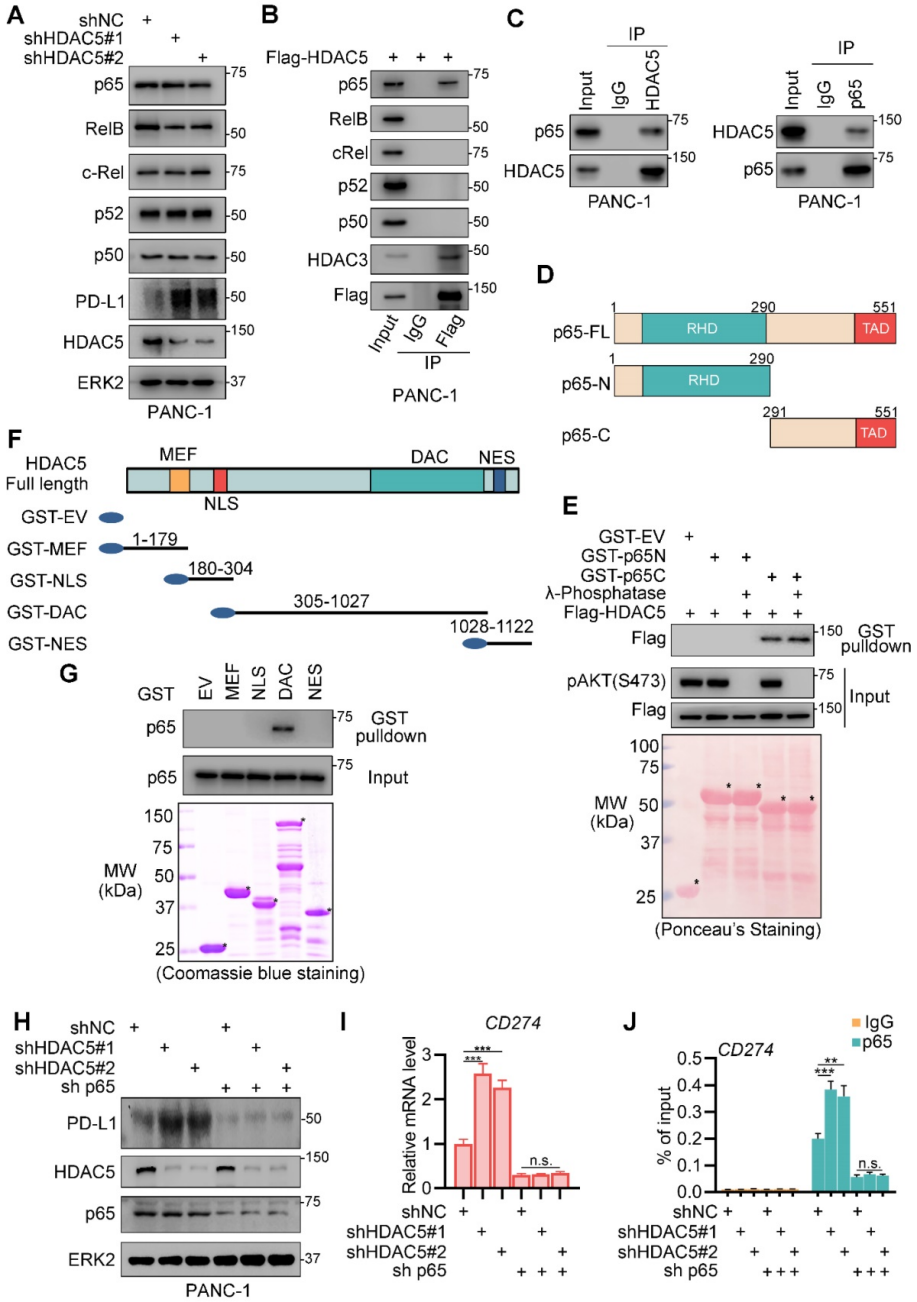
** HDAC5 interacts with p65. (A)** PANC-1 cells were infected with lenti-virus expressing indicated shRNAs for 48 h. After a 48 h puromycin selection, cells were harvested for western blot analysis. **(B)** PANC-1 cells were transfected with indicated plasmid. 48 h after transfection, cells were harvested for co-IP assay. **(C)** PANC-1 cells were harvested for reciprocal co-IP of HDAC5 and p65. **(D)** Schematic diagram depicting a set of GST-p65 recombinant protein constructs. **(E)** Western blot analysis of Flag-HDAC5 proteins in PANC-1 whole cell lysate (WCL) pulled down by GST or GST-p65 recombinant proteins under the condition with or without λ-phosphatase treatment. **(F)** Schematic diagram depicting a set of GST-HDAC5 recombinant protein constructs. **(G)** Western blot analysis of p65 proteins in PANC-1 WCL pulled down by GST or GST-HDAC5 recombinant. **(H-J)** PANC-1 cells were infected with lenti-virus expressing indicated shRNAs for 48 h. After a 48 h puromycin selection, cells were harvested for western blot analysis (H), RT-qPCR (I) and ChIP-qPCR (J). Data are shown as mean ± SD (n = 3, n.s. not significant, ** *P* < 0.01, *** *P* < 0.001).

To investigate the underlying mechanism by which HDAC5 regulates NF-κB function, we checked the effects of HDAC5 knockdown on NF-κB/Rel family members. Although HDAC5 knockdown in PANC-1 cells slightly decreased RelB expression levels, it had no effect on the expression of any of the other proteins tested (Figure 3A). Co-immunoprecipitation (co-IP) analysis in PANC-1 cells showed that ectopically expressed HDAC5 interacted with p65 but not with other NF-κB/Rel family members (Figure 3B). This interaction between HDAC5 and p65 was confirmed at the endogenous level in pancreatic cancer cell line (Figure 3C). We also demonstrated that the C-terminal of p65 and the DAC domain of HDAC5 were essential for the interaction of HDAC5 with p65 (Figure 3D-G). Since phosphorylation is crucial for HDAC5 activation and cellular translocation 29, 30, we sought to determine whether HDAC5-p65 interaction is regulated by HDAC5 phosphorylation. Interestingly, λ-phosphatase treatment had no profound effects on the interaction between HDAC5 and GST-p65-C-terminus (Figure 3E), indicating that the HDAC5 phosphorylation status has no effect on the formation of the HDAC5-p65 complex.

To assess the relevance of p65 in the ability of HDAC5 to regulate PD-L1 expression, we generated silenced HDAC5 and p65 in PANC-1 cells. In p65-expressing cells, HDAC5 knockdown significantly enhanced the binding of p65 to the promoter region of *CD274* and upregulated PD-L1 expression (Figure 3H-J). In contrast, in the absence of p65, HDAC5 knockdown failed to upregulate PD-L1 expression. Consistently, the binding of p65 to the promoter region of *CD274* was unaltered (Figure 3H-J). These data indicated that HDAC5 directly interact with p65, repressing p65-dependent PD-L1 expression.

### Phosphorylation of p65 at serine-311 impedes HDAC5-p65 interaction

**Figure 4:**
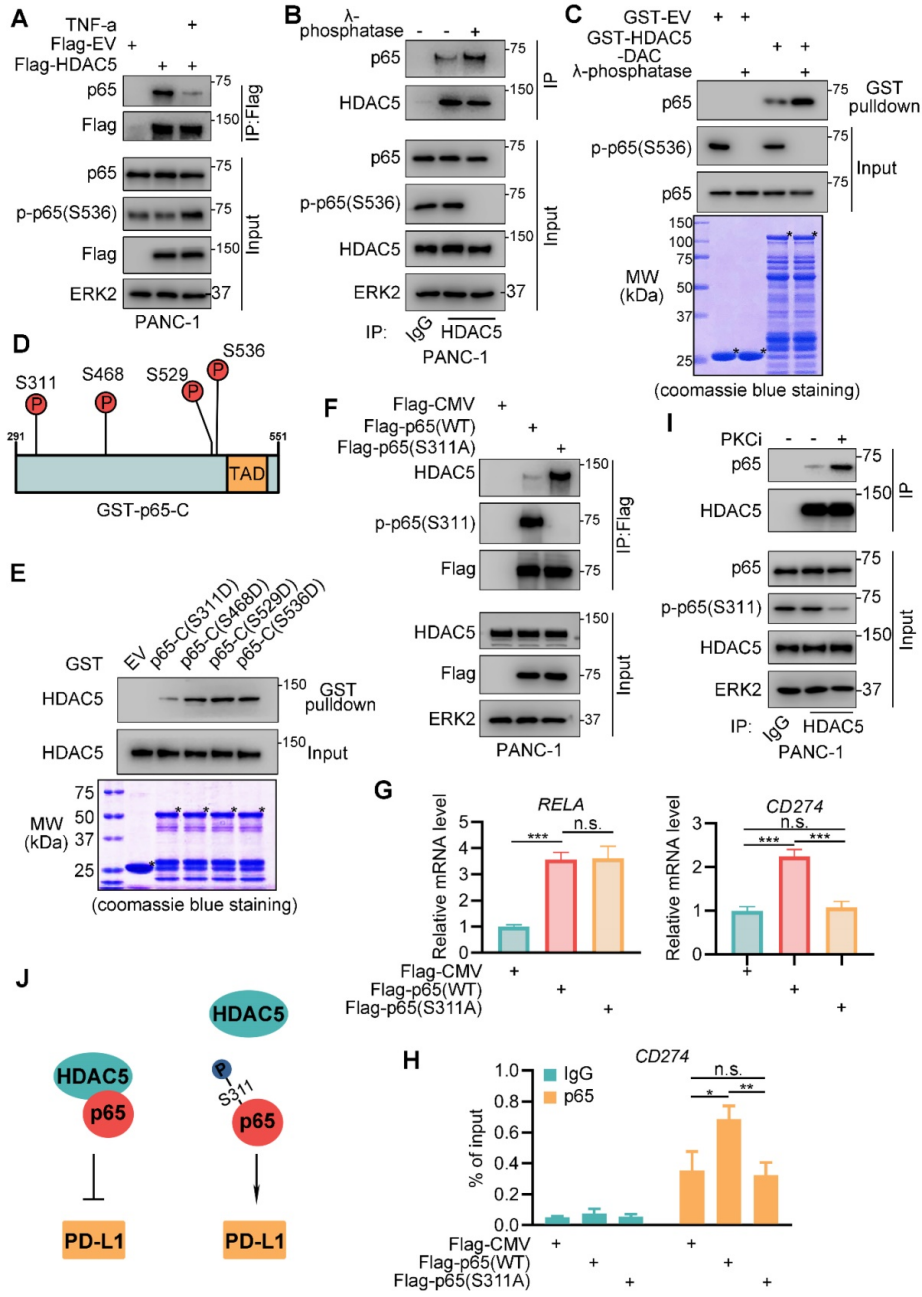
** Phosphorylation at serine-311 of p65 impedes HDAC5-p65 interaction. (A)** PANC-1 cells were infected with indicated plasmid for 24 h and treated with mock treatment (DMSO) or TNF-α for another 24 h. Then cells were harvested for co-IP assay. **(B)** WCL from PANC-1 cells were treated with or without λ-phosphatase, and then were applied to co-IP assay. **(C)** Western blot analysis of p65 protein from WCL of PANC-1 cells pulled down by GST or GST-HDAC5-DAC under the condition with or without λ-phosphatase treatment. **(D)** Schematic diagram depicting putative phosphorylation site on the C terminal of p65. **(E)** Western blot analysis of HDAC5 from WCL of PANC-1 pulled down by GST or indicated mutated GST-p65-C recombinant proteins. **(F-H)** PANC-1 cells were transfected with indicated plasmid for 48 h, and were harvested for co-IP assay (F), RT-qPCR (g) and ChIP qPCR (H). Data are shown as mean ± SD (n = 3, n.s. not significant, * *P* < 0.05, ** *P* < 0.01, *** *P* < 0.001). **(I)** PANC-1 cells were treated with indicated drugs (Staurosporine, 1 µM) or mock treatment (DMSO) for 24 h, and were harvested for co-IP assay. **(J)** Model depicting the mechanism that phosphorylation on S311 of p65 impedes HDAC5-p65 interaction and promote PD-L1 transcription.

The activation of NF-κB by TNF-α and other pro-inflammatory cytokines are highly implicated in inflammation and cancer 18. Here, we investigated the role of TNF-α on HDAC5-p65 interaction. Intriguingly, TNF-α treatment diminished HDAC5-p65 interaction (Figure 4A). As TNF-α activates NF-κB through MAP3K7-IKK signaling 18, we sought to determine the relevance of p65 phosphorylation in the ability of TNF-α to inhibit the formation of the HDAC5-p65 complex. λ-phosphatase treatment significantly increased HDAC5-p65 interaction in PANC-1 cells (Figure 4B). Similarly, λ-phosphatase treatment increased the ability of p65 to bind GST-HDAC5-DAC (Figure 4C). Nevertheless, HDAC5 phosphorylation has no effect on HDAC5-p65 binding (Figure 3E). Collectively, these data suggest that the phosphorylation of p65 but not HDAC5 interferes with HDAC5-p65 interaction.

The p65 C-terminus contains 4 phosphorylation sites, which may be implicated in response to TNF-α (Figure 4D) 31-35. These phosphorylation sites were mutated individually into aspartic acid (D) to mimic phosphorylation. GST pull-down revealed that in contrast to the other mutations, S311D decreased p65-C binding to HDAC5 (Figure 4E). Consistently, the S311 phosphorylation-resistant mutant (S311A) of p65 exhibited increased binding to HDAC5 compared with the wild-type (WT) p65 (Figure 4F). S311 of p65 is phosphorylated by PKCζ in response to TNF-α 36. Here, we found that pan PKC inhibitor, staurosporine, significantly enhanced the binding of HDAC5 to p65 (Figure 4I) and inhibited the expression of PD-L1 on mRNA and protein level (Figure S3A-B). Moreover, overexpression of WT Flag-p65 significantly enhanced the binding of p65 to the promoter region of *CD274,* as well as increased *CD274* mRNA levels; however, we did not observe these effects with the Flag-p65 (S311A) mutant (Figure 4G-H). These data suggest that TNF-α-induced S311 phosphorylation of p65 inhibits HDAC5-p65 interaction and HDAC5-mediated transcriptional repression of PD-L1 (Figure 4J).

### HDAC5 mediates p65 deacetylation at lysine-310

**Figure 5:**
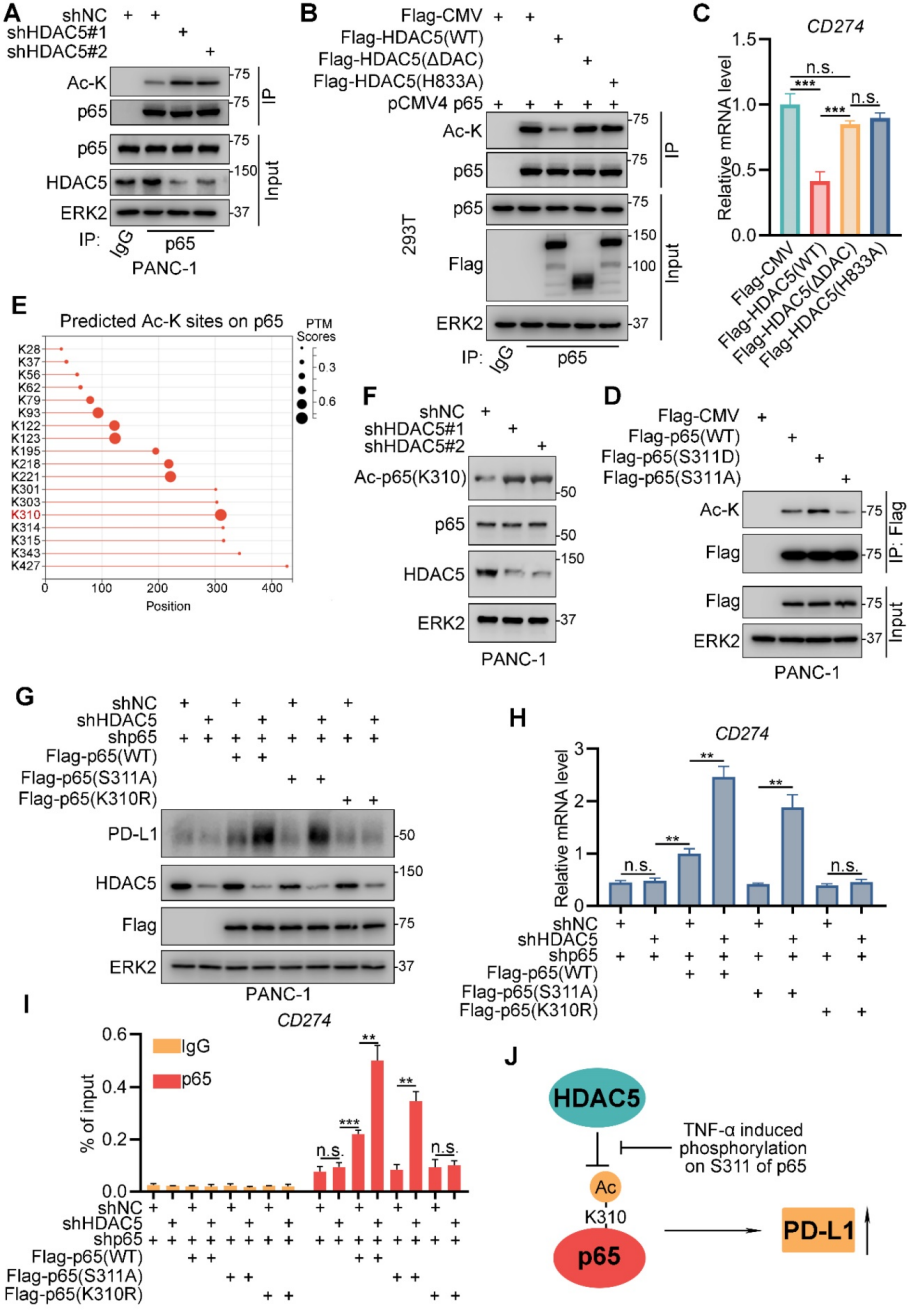
** HDAC5 deacetylates lysine-310 of p65. (A)** PANC-1 cells were infected with lenti-virus expressing indicated shRNAs for 48 h. After 48 h puromycin selection, cells were harvested for co-IP assay. **(B-C)** 293T cells were transfected with indicated plasmids for 48 h, and then were harvested for co-IP assay (B) and RT-qPCR (C). Data are shown as mean ± SD (n = 3, n.s. not significant, *** *P* < 0.001). **(D)** PANC-1 cells were transfected with indicated plasmids for 48 h and then were harvested for co-IP. **(E)** Diagram depicting the predicted acetylated lysine sites on p65 by MusiteDeep (https://www.musite.net/). **(F)** PANC-1 cells were infected with indicated lenti-virus for 48 h. After a 48 h puromycin selection, cells were harvested for western blot analysis. **(G-I)** PANC-1 cells were infected with lenti-virus expressing indicated shRNAs for 48 h. After a 48 h puromycin selection, cells were transfected with indicated plasmids for 48 h. Then, cells were harvested for western blot analysis (F), RT-qPCR (G) and ChIP-qPCR (H). Data are shown as mean ± SD (n = 3, n.s. not significant, ** *P* < 0.01, *** *P* < 0.001). **(J)** Schematic model depicting the mechanism that HDAC5 repress PD-L1 expression via deacetylates K310 of p65, while phosphorylation at S311 of p65 impedes this process.

HDAC5 has been shown to deacetylate numerous non-histone substrates, such as STAT3, HDAC2, and SOX9 37-39. Lysine acetylation of p65 enhances the transcriptional activity of NF-κB and is essential for p65 binding to BRD4 and other co-activators 40, 41. Here, we performed co-IP assays and found that HDAC5 silencing significantly increased the lysine acetylation levels of p65 (Figure 5A). Overexpression of WT HDAC5 decreased the lysine acetylation levels of p65. In contrast, HDAC5 lacking the DAC domain (HDAC5-ΔDAC) and the deacetylase-dead mutant HDAC5-H833A had no effect on p65 acetylation levels (Figure 5B). Consistently, WT HDAC5 overexpression decreased the mRNA levels of *CD274*, whereas HDAC5-ΔDAC and HDAC5-H833A had no effect on *CD274* mRNA levels (Figure 5C). Co-IP assays indicated that the p65 (S311D) mutation, which abrogates HDAC5 binding, had an increased acetylated lysine level to level comparable to WT p65 (Figure 5D). While the A mutant (p65-S311A) had a lower acetylated lysine level compared to WT p65 (Figure 5D). These data suggest that HDAC5 downregulates PD-L1 expression via p65 deacetylation and that phosphorylation of p65 at S311 alters HDAC5-p65 binding and p65 acetylation status.

To further confirmed the lysine that is deacetylated by HDAC5, we applied a deep-learning tool, 'MusiteDeep', to predict the lysine sites that might be acetylated on protein p65 (Figure 5E). There're 7 predicted sites on p65-C (291-551), the domain interacted with HDAC5 (Figure 5E). We generated p65-C acetylation-resistant mutants by individually mutating the 7 putative acetylated lysines into arginine (Figure S3C) 41, 42. Knockdown of HDAC5 failed to increase lysine acetylation levels of p65-K310R mutant but not the other 6 mutants (K301R, K303R, K314R, K315R, K343R and K427R) (Figure S3C), suggesting that HDAC5 specifically deacetylates K310 of p65. Moreover, the knock-down of HDAC5 resulted in a prominent increase of the level of acetyl-p65 (K310) which was detected by the site-specific acetylation antibody (Figure 5F). We also observed consistent phenomenon that the treatment of PKC inhibitor, which promote the formation of HDAC5-p65 complex, decreased the lysine acetylation level of WT p65, but not p65-K310R mutant (Figure S3D). Rescue with WT p65 significantly upregulated the mRNA and protein levels of PD-L1 regardless of the HDAC5 status (Figure 5G-I; Figure S4A). The p65 (S311A) mutant, which is easily bind to HDAC5 and deacetylated by HDAC5, failed to make a difference under the HDAC5 proficient status. When HDAC5 was silenced, p65-S311A could bind the *CD274* promoter and induce its expression (Figure 5G-I; Figure S4A). However, the acetylation-resistant mutant p65-K310R failed to do so, regardless of the HDAC5 status (Figure 5G-I; Figure S4A). These data suggest that HDAC5 deacetylates p65 at K310, inhibiting p65 binding to the promoter region of *CD274* and reducing PD-L1 levels. TNF-α interferes with this process by promoting p65 phosphorylation at S311 (Figure 5J).

Since HDAC5 knockdown resulted in the enhanced recruitment of p65 on the promoter of *CD274*. We were interest if K310 acetylation on p65 regulate its cellular location or HDAC5 knock-down activated p65 upstream signaling. However, the knock-down of HDAC5 didn't affect the cellular location of p65 (Figure S4B), nor the level of the upstream signaling of p65 like IKβα, IKKα/β and phospho-IKKα/β (Figure S4C). Next, we sought to determine if HDAC5 mediated deacetylation regulates p65's interaction with important co-activator like BRD4 43. Co-IP assay indicated that HDAC5 knock-down prominently increased the binding between p65 and BRD4 (Figure S4D), and ChIP-qPCR showed that HDAC5 knock-down lead to a significant elevation of BRD4 enrichment at the promoter of *CD274* (Figure S4E). Consistently, the acetylation-resistant mutant p65-K310R had minimal ability to interact with BRD4 compared to WT p65 (Figure S4F). Based on these data, we thought it's a feasible explanation that HDAC5 mediated deacetylation on p65 inhibited its interaction with co-activators like BRD4, and further decreased p65's chromatin occupancy at the locus of its target genes like *CD274*.

### HDAC5 inhibition augments response to anti-PD1 therapy

Given the role of HDAC5 in modulating the expression PD-L1 in pancreatic cancer cells, we next assessed the potential clinical benefit of HDAC5 inhibition in pancreatic cancer patients treated with ICB. Consistent with our findings in human pancreatic cancer cells, *Hdac5* silencing in Panc02 murine pancreatic cancer cells upregulated Pd-l1 (Figure S5A-B). Panc02 cells infected with shControl or shHdac5 lentiviruses were subcutaneously injected into immunocompetent C57BL/6 mice. Subsequently, mice were treated with anti-PD-1 antibody or IgG control antibody (Figure S5C). Interestingly, *Hdac5* silencing enhanced tumor growth (Figure S5D). Furthermore, anti-PD-1 treatment had a limited effect on the growth of shControl tumors. In the *Hdac5* knockdown group, anti-PD-1 treatment strongly suppressed tumor growth (Figure S5D). Anti-PD-1 treatment moderately increased the tumor infiltration of CD45^+^CD8^+^ T cells and CD45^+^CD4^+^ T cells and decreased the infiltration of CD11b^+^Gr1^+^ myeloid cells and CD4^+^FOXP3^+^ Treg cells in shControl mice (Figure S5E; Figure S6E-F). In the Hdac5 knockdown group, anti-PD-1 treatment dramatically increased the tumor infiltration of CD45^+^CD8^+^ and CD45^+^CD4^+^ T cells as well as decreased the levels of CD11b^+^Gr1^+^ myeloid cells and CD4^+^Foxp3^+^ Treg cells in tumors (Figure S5E; Figure S6E-F).

Since HDAC5 silencing resulted in a vulnerability to ICB therapy, we sought to determine if HDAC5 inhibitor, LMK235, would sensitize PDAC to anti-PD1 treatment. The treatment of LMK235 significantly increased the expression of PD-L1 (Figure S5F-G), the acetylated lysine level of p65 (Figure S5H), and the p65 enrichment at the promoter of *CD274* (Figure S5I). Consistently, the combination of LMK235 with anti-PD-1 significantly increased the levels of tumor-infiltrating T cells (Figure S5J).

**Figure 6:**
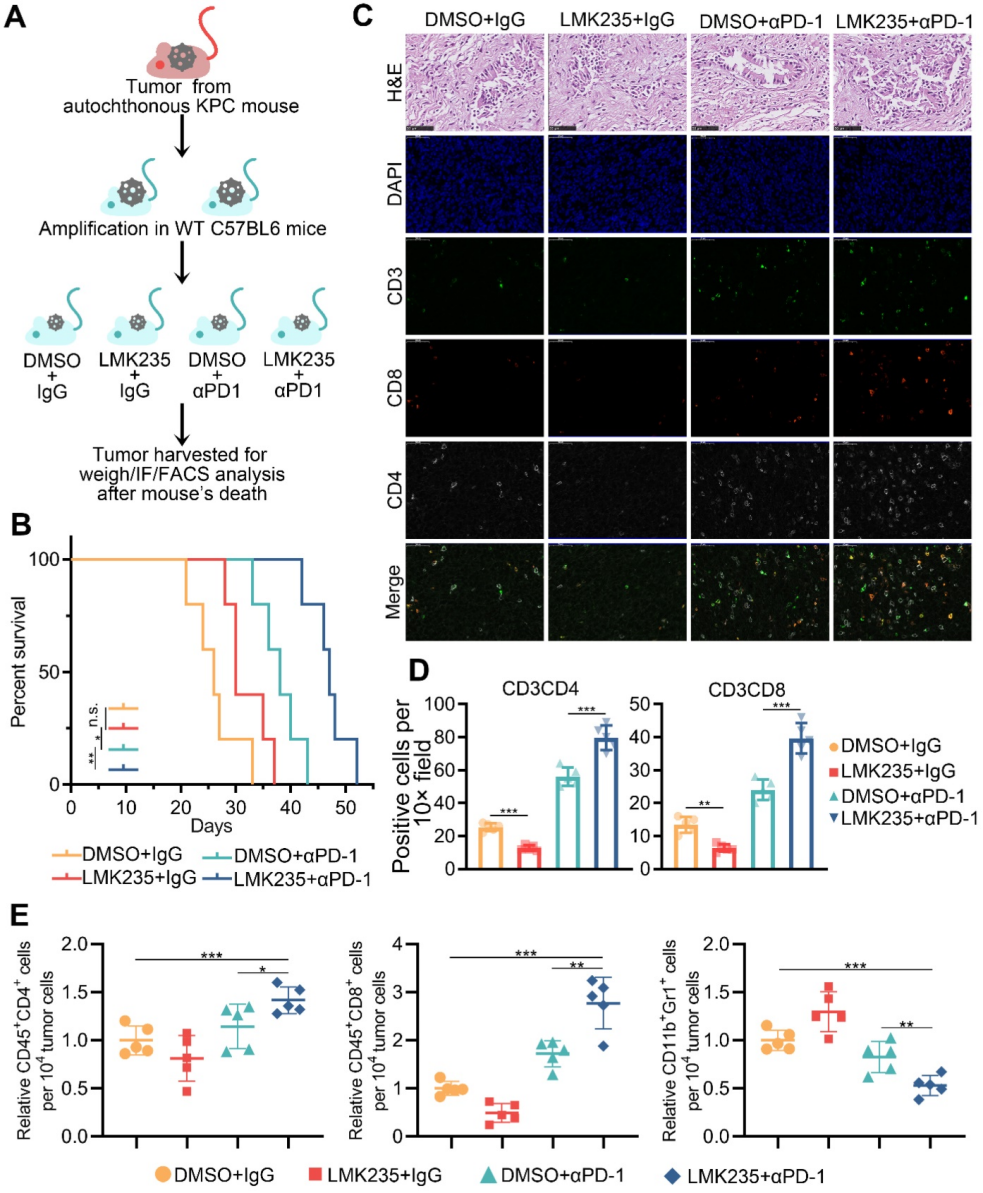
** HDAC5 inhibition confers the vulnerability to anti-PD1 therapy. (A)** Schematic diagram depicting the treatment plan for mice bearing KPC derived tumor. **(B)** Kaplan-Meier survival curves for each treatment group. * *P* < 0.05, ** *P* < 0.01 (Gehan-Breslow-Wilcoxon test). **(C-D)** Representative images of H&E staining and immunofluorescence staining of tumor samples in each group (C), and the quantification data (D). **(E)** FACS analysis of tumor infiltrated CD45+CD8+ T cells, CD45+CD4+ T cells, and CD11b+Gr1+ myeloid cells in indicated treatment group. Data are shown as mean ± SD (n = 5), * *P* < 0.05, ** *P* < 0.01, *** *P* < 0.001.

To further investigate the role of HDAC5 in a model mimicking the mutational burden in human PDAC, we generated a mouse PDAC model using genetically engineered KPC mouse (*Kras*^G12D/+^; *LSLTrp53*^R172H/+^; *Pdx-1-Cre*) derived tumor cells (Figure 6A; Figure S6A-B). The co-treatment of LMK235 and anti-PD-1 prolonged the survival of mice bearing KPC derived tumor (Figure 6B). Immunofluorescence staining of tumor samples and FACS analysis indicated the increased infiltrating T cells and decreased Treg cells in co-treatment group (Figure 6C-E; Figure S6C-D). Taken together, our data indicated the synergistic effect of HDAC5 inhibition and anti-PD-1 treatment in immunotherapy resistant pancreatic cancer.

## Discussion

**Figure 7:**
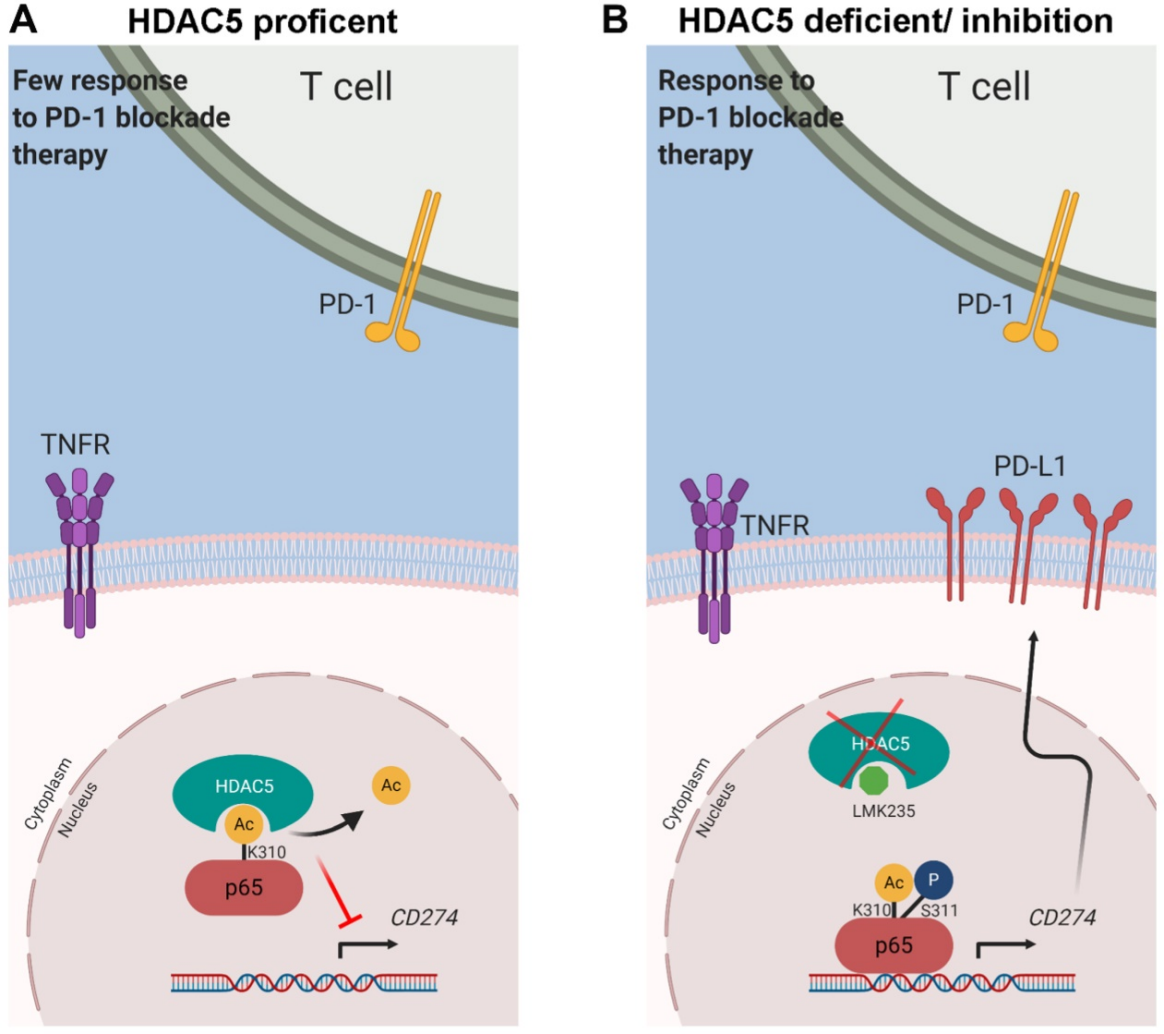
** Models depicting the mechanism that HDAC5 modulates PD-L1 expression via p65 deacetylation. (A)** In HDAC5 proficient cells, HDAC5 mediated the deacetylation on K310 of p65, decreasing the p65 enrichment at the gene loci of *CD274*, repressing the expression of PD-L1 and contributing to the situation of resistance to ICB in PDAC. **(B)** In the situation of HDAC5 silencing or inhibition, acetylated p65 was able to enriched at the gene loci of *CD274*, promoting the expression of PD-L1 and resulting in a relatively favorable response to ICB therapy.

Reversible acetylation of p65 regulates its cellular location, DNA binding ability, and transcriptional activity. p65 contains multiple lysines that are acetylation targets, and acetylation on different sites regulates distinct biological activities 41. For instance, acetylation at K218 and K221 inhibits p65 binding to IκBα, and K221 acetylation regulates p65 nuclear accumulation. K310 acetylation has been identified to regulate the transcriptional activity of p65 41. p65 K310 can be acetylated by p300/CBP 44, enhancing the binding of p65 to co-activators including BRD4, thereby promoting the maintain of constitutive activation of NF-κB 40. HDAC5 and other HDACs were reported to deacetylate a variety of non-histone substrates 37-39. However, class IIa HDAC members are thought to have limited enzymatic activity due to the amino acid substitution in the catalytic center 14. They might act as a scaffold protein and work with other enzyme-active HDACs like HDAC3 to complete the process of deacetylation 45. To verify this possibility, we knocked-down HDAC3 and didn't observe significant alteration on the lysine acetylation level of p65 (Figure S4G-H). A plausible explanation is that HDAC5 work as an acetylation reader and cooperation with multiple HDACs. The effect of HDAC3 knock-down might be compensated by other HDACs. While this deduction warrants more investigation. In this study, we identified the irreplaceable role of HDAC5 in the process of K310 deacetylation, the 'Achilles' Heel' of p65, repressing the transcriptional activity of NF-κB and inhibiting the expression of PD-L1 (Figure 7A).

HDACs are important deacetylases which can be categorized into 4 groups according to their yeast homologs, Class I (HDAC1, 2, 3 and 8), Class II (4, 5, 6, 7, 9 and 10), Class III (Sirtuins) and Class IV (HDAC11) 46. And Class II HDACs can be further subdivided into two groups, Class IIa (HDAC4, 5, 7 and 9) and Class IIb (HDAC6 and 10). Tons of studies have observed the diversified function of different HDACs. The Class IIb member, HDAC6, even exhibited contradictory activities to HDAC5 in the regulation of tumor immunity 47. Class IIa members were thought to be distinct to other HDACs in terms of molecular weight and the limited enzymatic activity 14. Furthermore, our previous research found that HDAC5 work with retinoblastoma protein (RB) through a completely different binding motif compared to that of Class I HDACs 48. This phenomenon also helps us to explain the diversity of different HDACs. More interestingly, HDAC5 itself also plays different roles in different tumors 15, 16. And its function will also change due to the different subtypes in the same tumor, ER status determines the effect of HDAC5 on the vulnerability to CDK4/6 inhibitors in breast cancer 48. This complexity also indicated that more investigation is needed to clarify the underlying regulatory mechanism.

The early activation of NF-κB is regulated by IKK-mediated phosphorylation of IκB proteins 21. Upon phosphorylation, IκB is degraded via the proteasome pathway, liberating NF-κB protein, which then translocated into the nucleus 49, 50. Phosphorylation of p65 and other NF-κB subunits by IKK and other kinases positively regulates the transcriptional activity of NF-κB 35. S311 in the N-terminal Rel homology domain (RHD) of p65 is phosphorylated by PKCζ in response to TNF-α 36. This phosphorylation enhances p65 binding to CBP, which recruits RNA polymerase II to the promoter of target genes 35. NF-κB activation defects have been reported in TNF-α-stimulated PKCζ^-/-^ mouse embryonic fibroblasts, despite the unaltered IKK function 51. In this study, we demonstrated that p65 phosphorylation at S311 promotes NF-κB activation by inhibiting the interaction of HDAC5 with p65. Intriguingly, anergic CD8^+^ T cells with inactive NF-κB exhibited the lack of p65 phosphorylation and acetylation at S311 and K310, respectively 52. Our data provide a plausible explanation for this phenomenon since p65 phosphorylation at S311 inhibited HDAC5-mediated deacetylation of p65 K310. Thus, our data underpin an unappreciated model where the phosphorylation of p65 protects the acetylated lysine, and these 2 post-translational modifications work together to maximize NF-κB activation (Figure 7B).

Combined treatment with chloroquine and chemotherapy has been reported to sensitize pancreatic tumors to immunotherapy 3, 53. Chloroquine-mediated inhibition of autophagy protected MHC-I molecules from lysosomal degradation 3. Additionally, chemotherapeutic agents have been shown to upregulate PD-L1 expression via NF-κB signaling pathway 53. Consistent with this finding, we provided evidence that HDAC5 is a pivotal negative regulator of NF-κB activity. HDAC5 silencing or inhibition augmented the ability of anti-PD-1 therapy to increase the levels of tumor-infiltrating CD8^+^ T cells and prolong the survival of tumor-bearing mice (Figure 7B). Thus, our findings support that HDAC5 inhibition may improve the clinical benefit of immunotherapy targeting PD-1/PD-L1 in PDAC patients. Additionally, HDAC5 may represent a useful biomarker predicting ICB response in PDAC patients.

In conclusion, we report a previously uncharacterized role of HDAC5 in enhancing tumor immunity by repressing NF-κB-mediated PD-L1 expression. Particularly, HDAC5 inhibits the transcriptional activity of NF-κB by directly binding to p65 and mediating p65 K310 deacetylation; p65 phosphorylation at S311 interferes with this process. These findings provide a strong rationale for targeting HDAC5 to restore ICB sensitivity in immunotherapy-resistant pancreatic tumors.

## Supplementary Material

Supplementary figures and tables.
