# Supplementary material for: HDAC5 modulates PD-L1 expression and cancer immunity via p65 deacetylation in pancreatic cancer
Source: Theranostics. 2022 Jan 31;12(5):2080–94. doi: 10.7150/thno.69444 (PMC8899586; doi:10.7150/thno.69444)
Supplement: Supplementary file 1 — Supplementary figures and tables. [file thnov12p2080s1.pdf]

Supplementary materials for

HDAC5 modulates PD-L1 expression and cancer immunity via the deacetylation of p65 in pancreatic cancer

Yingke Zhou, Xin Jin, Haixin Yu, Gengdu Qin, Penglin Pan, Jingyuan Zhao, Taoyu Chen, Xueyi Liang, Yan Sun, Bo Wang, Dianyun Ren, Shikai Zhu, Heshui Wu

The file includes:

- Supplementary figures and figure legends
- Supplementary Table S1. Sequence of primers and gene specific shRNAs
- Supplementary Table S2. Information of antibodies
- Supplementary Table S3. Information of chemicals
- Supplementary Table S4. Information of recombinant DNA
- Supplementary Table S5. TCGA samples included in this study
- Supplementary Table S6. Top20 Pathways negatively correlated with HDAC5 expression in the GSEA of TCGA pancreatic cancer dataset

Supplementary figures

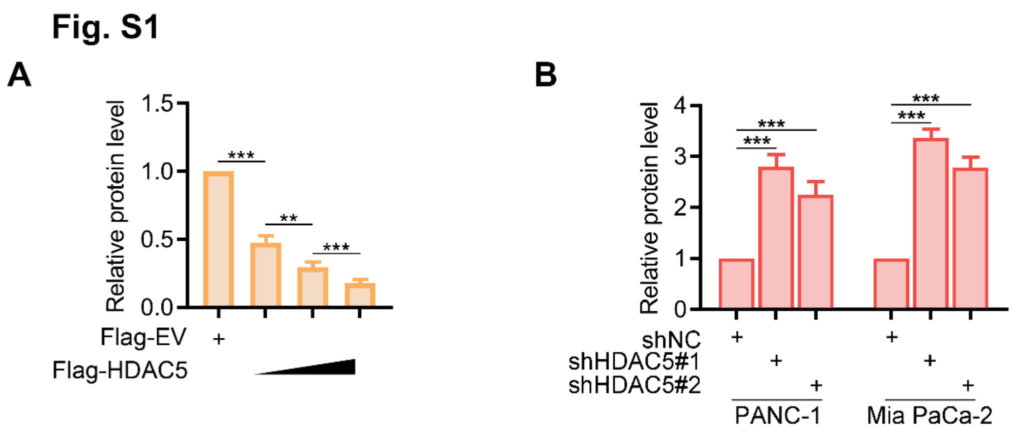

Figure S1 Supplementary data to Figure 1

(A) Histogram showing the relative protein level of PD-L1 in three independent replicates of the assay in Figure 1G. **(B)** Relative protein level of PD-L1 in Figure 1I (n= 3).

Fig. S2

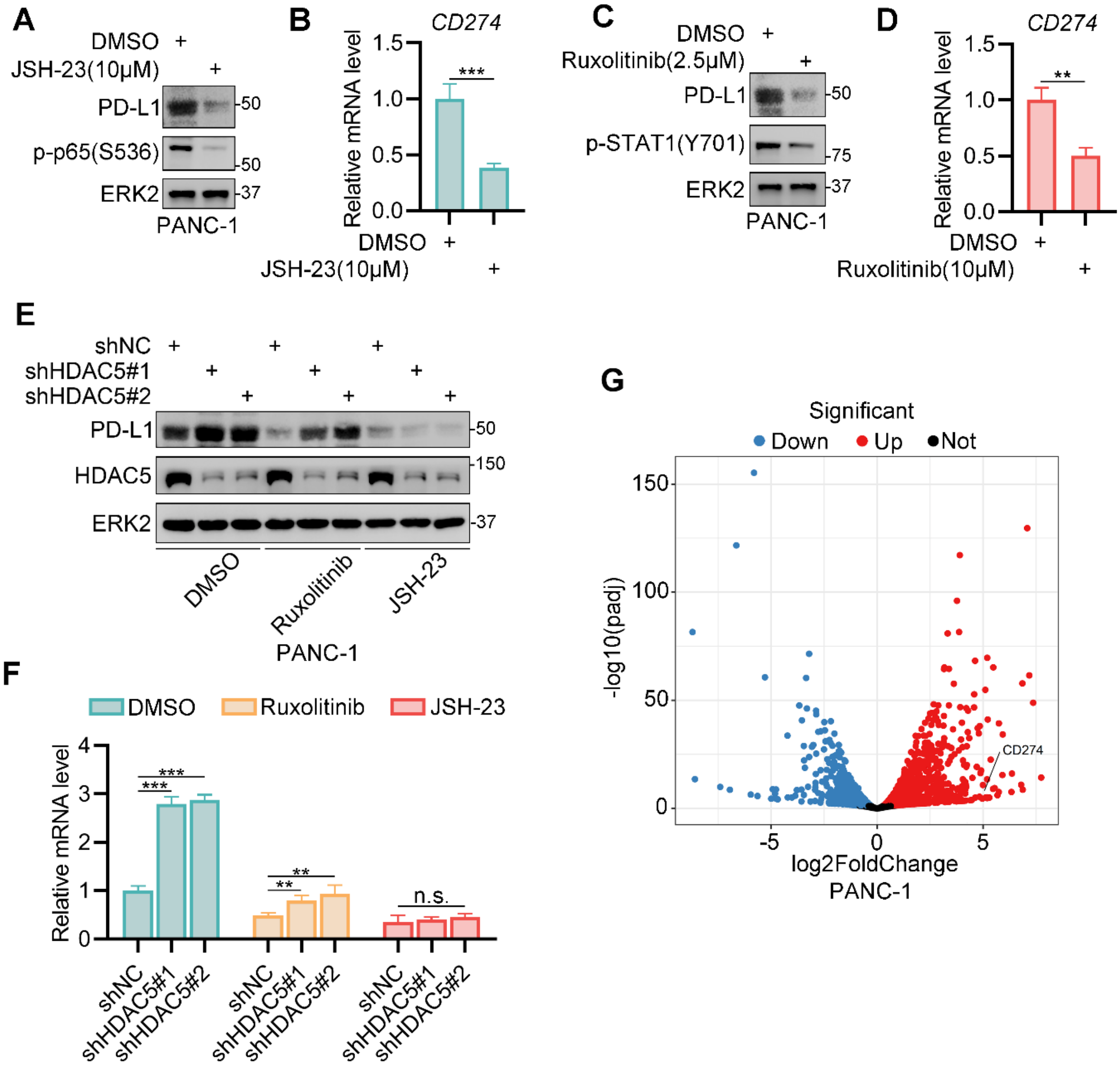

Figure S2 Supplementary data to Figure 2

(A-D) PANC-1 cells were treated with indicated chemicals for 24 h, and then were harvest for western blot (A, C) and RT-qPCR, data are shown as mean ± SD (n= 3, ** *P* < 0.01, *** *P* < 0.001) (B, D). **(E-F)** PANC-1 cells were infected with lenti-virus expressing indicated shRNAs for 48 h. After a 48 h puromycin selection, cells were treated with indicated drugs for 24 h (2.5 μM Ruxolitinib, 10 μM JSH-23). Then cells were harvested for western blot (E) and RT-qPCR. Data are shown as mean ± SD (n = 3, n.s. not significant, ** *P* < 0.01) (F). **(G)** Volcano plot depicting the differentially expressed genes (DEGs) in HDAC5 knock-down PANC-1 cells compared to control cells via RNA-seq.

Fig. S3

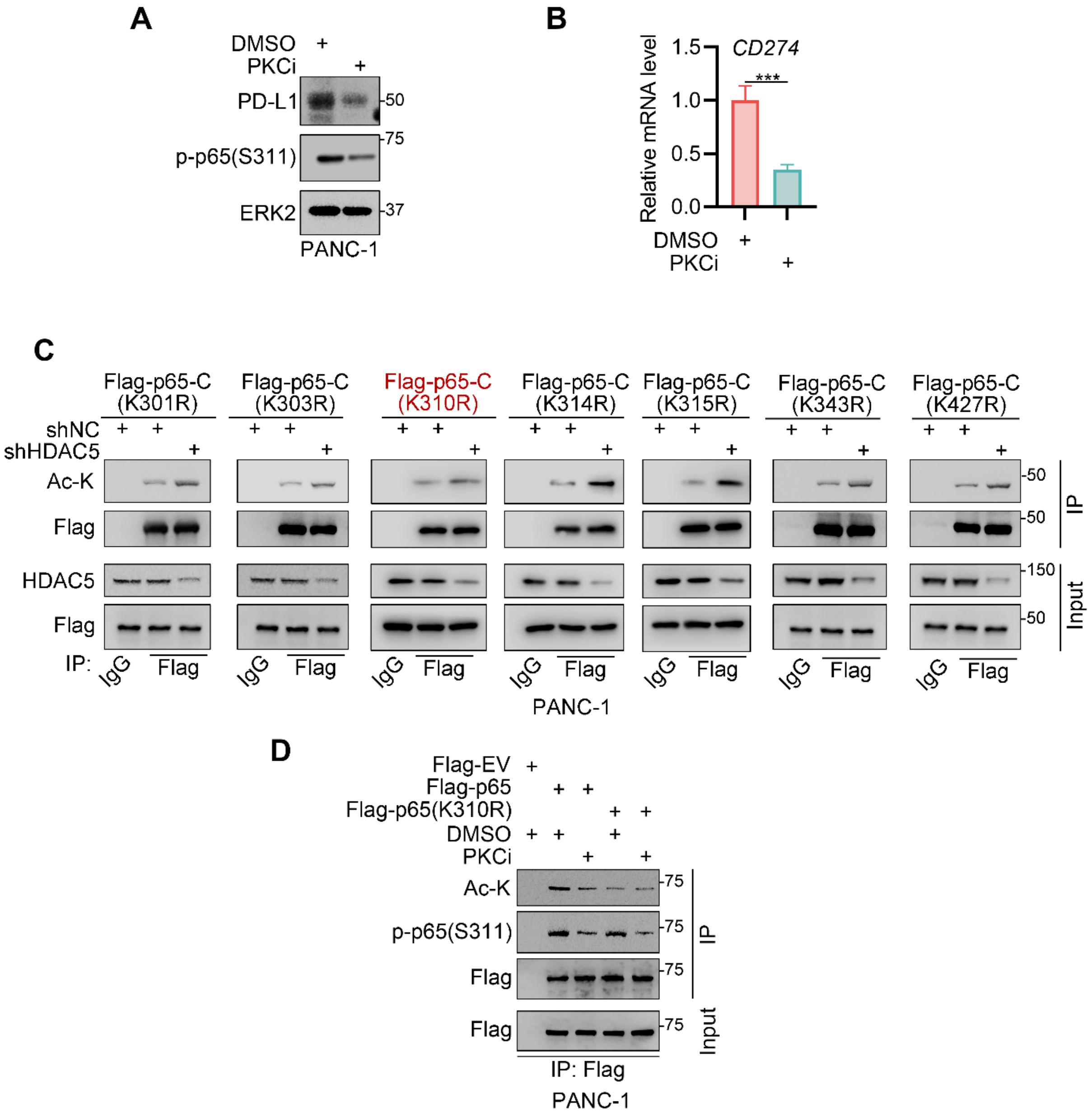

Figure S3 PKC inhibitor represses PD-L1 expression and p65 acetylation

(A-B) PANC-1 cells were treated with indicated drugs (Staurosporine, 1μM) for 24 h, and then were harvested for western blot (A) and RT-qPCR, data are shown as mean ± SD (n= 3, *** $P < 0.001$) (B). **(C)** PANC-1 cells were transfected with indicated plasmids for 48 h, then cells were harvested for co-IP assay. **(D)** PANC-1 cells were transfected with indicated plasmids, 24 h after transfection, cells were treated with indicated drugs for 24 h, and then were harvest for co-IP assay.

Fig. S4

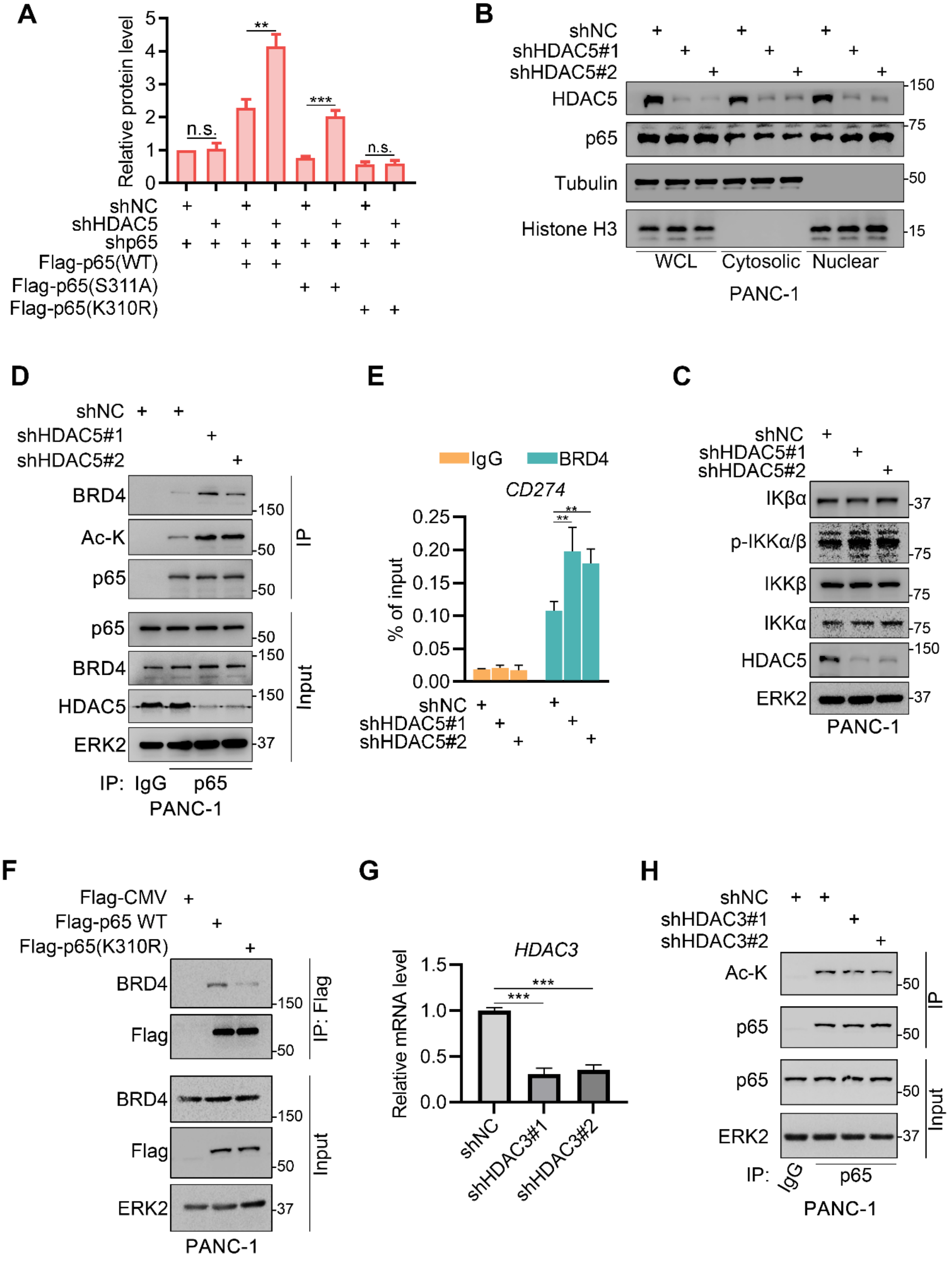
A
Relative protein level
n.s.
**
***
n.s.
shNC
shHDAC5
shp65
Flag-p65(WT)
Flag-p65(S311A)
Flag-p65(K310R)
B
shNC
shHDAC5#1
shHDAC5#2
HDAC5
p65
Tubulin
Histone H3
WCL
Cytosolic
Nuclear
PANC-1
D
shNC
shHDAC5#1
shHDAC5#2
BRD4
Ac-K
p65
IP
p65
BRD4
HDAC5
ERK2
Input
IP: IgG p65
PANC-1
E
IgG
BRD4
CD274
% of input
**
**
shNC
shHDAC5#1
shHDAC5#2
C
shNC
shHDAC5#1
shHDAC5#2
IKβα
p-IKKα/β
IKKβ
IKKα
HDAC5
ERK2
PANC-1
F
Flag-CMV
Flag-p65 WT
Flag-p65(K310R)
BRD4
Flag
IP: Flag
BRD4
Flag
ERK2
Input
PANC-1
G
HDAC3
Relative mRNA level
***
***
shNC
shHDAC3#1
shHDAC3#2
H
shNC
shHDAC3#1
shHDAC3#2
Ac-K
p65
IP
p65
ERK2
Input
IP: IgG p65
PANC-1

Figure S4 Acetylation on K310 of p65 promotes its binding with BRD4

(A) Relative protein level of PD-L1 in Figure 5F (n= 3). **(B)** Western blot analysis of whole cell lysate (WCL), cytosolic fractionation and nuclear fractionation from PANC-1 cells after the knock-down of HDAC5. **(C-E)** PANC-1 cells were infected with indicated lenti-virus, after a 48 h puromycin selection, cells were harvested for western blot (C), co-IP (d) and ChIP-qPCR (E), data are shown as mean ± SD (n= 3, ** $P < 0.01$). **(F)** PANC-1 cells were transfected with indicated plasmids for 48 h, and cells were harvested for co-IP analysis. **(G-H)** PANC-1 cells were infected with lenti-virus expressing indicated shRNAs, and after a 48 h puromycin selection, cells were harvested for RT-qPCR (G) and co-IP analysis (H). Data are shown as mean ± SD (n= 3, *** $P < 0.001$).

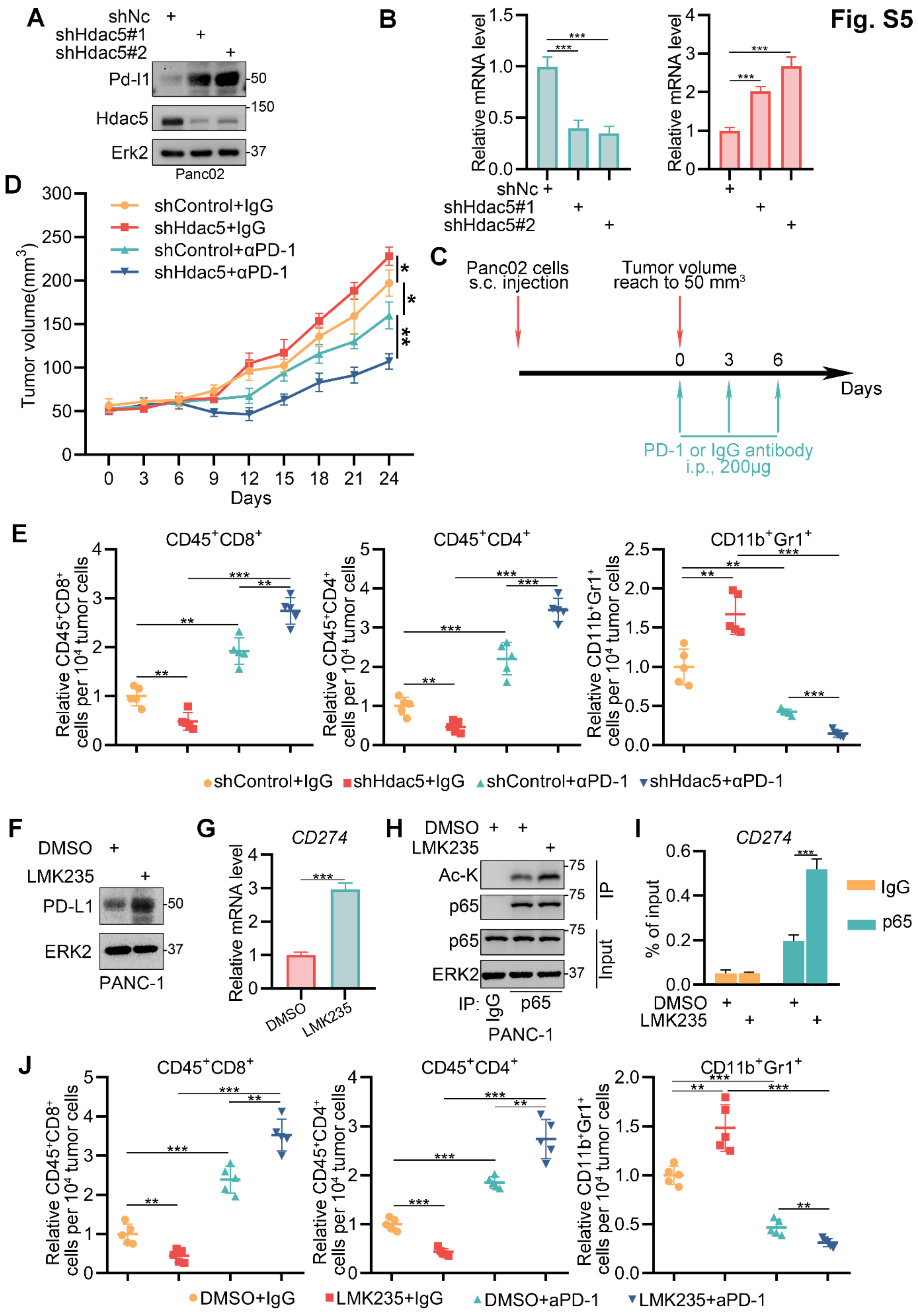
Fig. S5
A
shNc
shHdac5#1
shHdac5#2
Pd-l1
Hdac5
Erk2
Panc02
B
Relative mRNA level
shNc
shHdac5#1
shHdac5#2
D
Tumor volume(mm3)
Days
shControl+IgG
shHdac5+IgG
shControl+αPD-1
shHdac5+αPD-1
C
Panc02 cells s.c. injection
Tumor volume reach to 50 mm3
Days
PD-1 or IgG antibody i.p., 200μg
E
CD45+CD8+
CD45+CD4+
CD11b+Gr1+
Relative CD45+CD8+ cells per 10^4 tumor cells
Relative CD45+CD4+ cells per 10^4 tumor cells
Relative CD11b+Gr1+ cells per 10^4 tumor cells
shControl+IgG
shHdac5+IgG
shControl+αPD-1
shHdac5+αPD-1
F
DMSO
LMK235
PD-L1
ERK2
PANC-1
G
CD274
Relative mRNA level
DMSO
LMK235
H
DMSO
LMK235
Ac-K
p65
p65
ERK2
IP
Input
IP:
IgG
p65
PANC-1
I
CD274
% of input
IgG
p65
DMSO
LMK235
J
CD45+CD8+
CD45+CD4+
CD11b+Gr1+
Relative CD45+CD8+ cells per 10^4 tumor cells
Relative CD45+CD4+ cells per 10^4 tumor cells
Relative CD11b+Gr1+ cells per 10^4 tumor cells
DMSO+IgG
LMK235+IgG
DMSO+aPD-1
LMK235+aPD-1

Figure S5 HDAC5 silence or inhibition sensitize panc-02 derived PDAC mouse model to the treatment of α-PD1

(A-B) Panc 02 cells were infected with lentivirus expressing indicated shRNAs for 48 h. After 48 h puromycin selection, cells were harvested for western blot analysis (A) and RT-qPCR (B). Data are shown as mean ± SD ($n = 3$, *** $P < 0.001$). **(C)** Schematic diagram depicting the treatment plan for mice bearing subcutaneous Panc 02 tumors. **(D)** Growth curve of tumors in different groups. Data are shown as mean ± SD ($n = 5$), * $P <0.05$, ** $P < 0.01$. **(E)** At the end of the treatment, the numbers of CD45+CD8+ T cells, CD45+CD4+ T cells, and CD11b+Gr1+ myeloid cells that infiltrated in tumors in different treatments were analyzed by FACS. Data are shown as mean ± SD ($n = 5$), ** $P <0.01$, *** $P < 0.001$. **(F-G)** PANC-1 cells were treated with indicated drugs for 24 h, and cells were harvested for western blot (f) and RT-qPCR, data are shown as mean ± SD ($n= 3$, *** $P < 0.001$) (g). **(H-I)** PANC-1 cells were treated with indicated drugs for 24 h, and cells were harvested for co-IP (H) and ChIP-qPCR, data are shown as mean ± SD ($n= 3$, *** $P < 0.001$) (I). **(J)** FACS analysis of tumor infiltrated CD45+CD8+ T cells, CD45+CD4+ T cells, and CD11b+Gr1+ myeloid cells in indicated treatment group.

Fig. S6

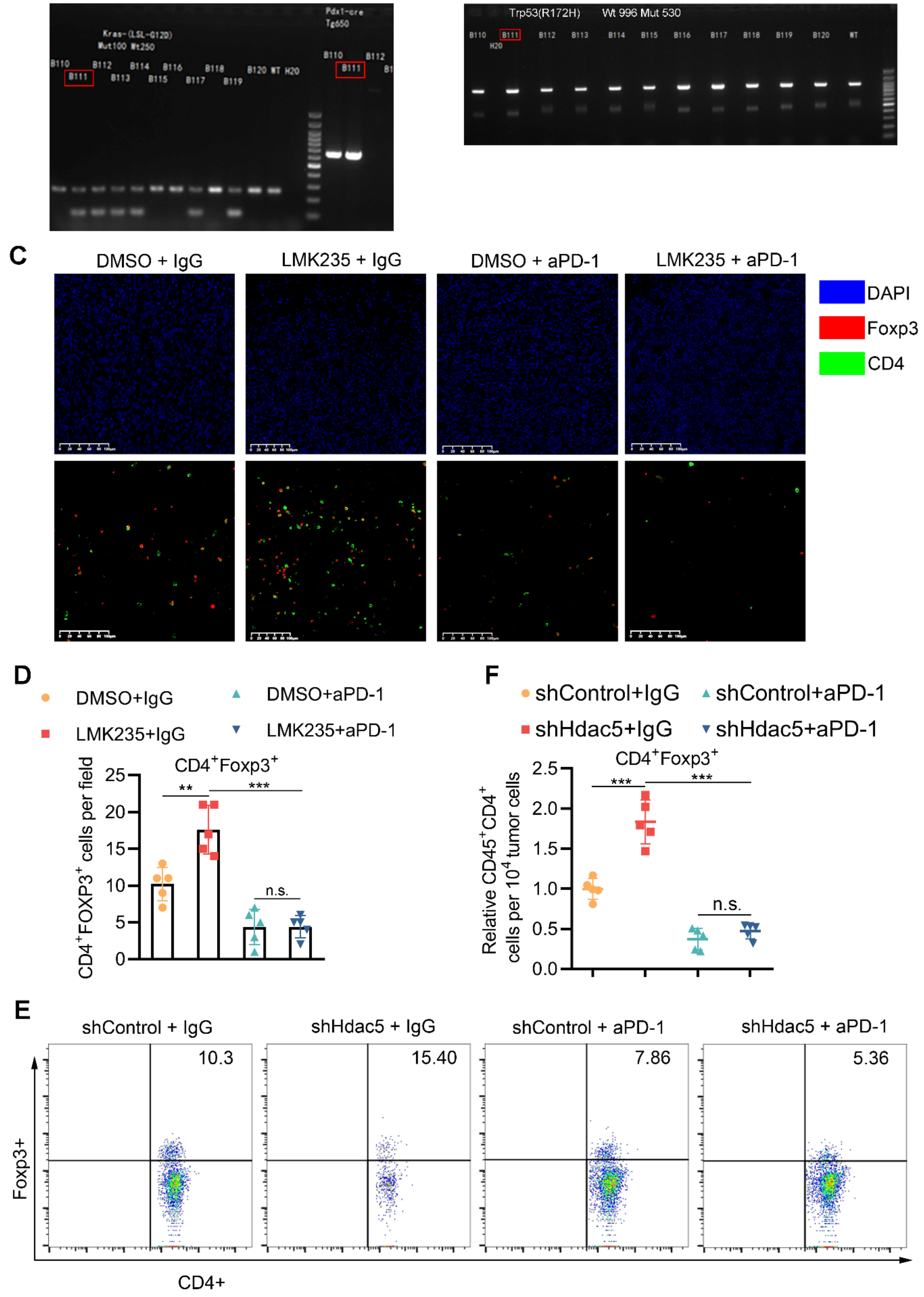
A
Kras-(LSL-G12D)
Mut100 Wt250
Pdx1-cre
Tg650
B110
B111
B112
B113
B114
B115
B116
B117
B118
B119
B120
WT
H20
B
Trp53(R172H)
Wt 996 Mut 530
C
DMSO + IgG
LMK235 + IgG
DMSO + aPD-1
LMK235 + aPD-1
DAPI
Foxp3
CD4
D
DMSO+IgG
LMK235+IgG
DMSO+aPD-1
LMK235+aPD-1
CD4+Foxp3+
CD4+FOXP3+ cells per field
n.s.
F
shControl+IgG
shHdac5+IgG
shControl+aPD-1
shHdac5+aPD-1
Relative CD45+CD4+ cells per 10^4 tumor cells
E
shControl + IgG
shHdac5 + IgG
shControl + aPD-1
shHdac5 + aPD-1
10.3
15.40
7.86
5.36
Foxp3+
CD4+

Figure S6 Supplementary data to mouse work

(A-B) Genotyping of the autochthonous KPC mouse (*Kras*$^{G12D/+}$; *LSLTrp53*$^{R172H/+}$; *Pdx-1-Cre*) used in Figure 6. **(C-D)** Representative images of immunofluorescence staining of tumor samples in indicated group (C), and the quantification data (D). Data are shown as mean ± SD (n = 5), n.s. not significant, ** $P < 0.01$, *** $P < 0.001$. **(E-F)** FACS analysis of tumor infiltrated $CD4^{+}Foxp3^{+}$ Treg cells in indicated groups (E) and quantification data (F). Data are shown as mean ± SD (n = 5), n.s. not significant, *** $P < 0.001$.

Table S1. Sequence of primers and gene specific shRNAs

Gene	Usage	Forward	Reverse
GAPDH	RT-qPCR	ACCCAGAAGACTGTGGATGG	TTCAGCTCAGGGATGACCTT
CD274	RT-qPCR	GGTGCCGACTACAAGCGAAT	AGCCCTCAGCCTGACATGTC
RELA	RT-qPCR	TGGCCCCTATGTGGAGATCA	GTATCTGTGCTCCTCTCGCC
HDAC5	RT-qPCR	CTGCGGAACAAGGAGAAGAG	GGGAACTCTGGTCCAAAGAA
HDAC3	RT-qPCR	CCTTTTCCAGCCAGTCATCAG	AACATTTCGGACAGTGTAGCC
Gapdh	RT-qPCR	AGGTTGTCTCCTGCGACTTCA	GGGTGGTCCAGGGTTTCTTACT
Hdac5	RT-qPCR	GACCAACCCACTGTGGTGAA	TCCAGTGTGGCTTTACGACC
Cd274	RT-qPCR	AATGCTGCCCTTCAGATCAC	ATAACCCTCGGCCTGACATA
CD274 promoter	ChIP-qPCR	GCACTTTAGGACGGAGGGTC	CTCTACTGCCCCCTAGACCA
CD274 enhancer	ChIP-qPCR	GGAGAGGCACTAAGAGGGAAA	AAGCATGAGGAATACGGAAGTCA

**shRNAs**	**Sequence**
sh-HDAC5-1	CCGGGACTGTTATTAGCACCTTTAACTCGAGTTAAAGGTGCTAATAACAGTCTTTTT
sh-HDAC5-2	CCGGGCTAGAGAAAGTCATCGAGATCTCGAGATCTCGATGACTTTCTCTAGCTTTTT
sh-p65-1	CCGGCGGATTGAGGAGAAACGTAAACTCGAGTTTACGTTTCTCCTCAATCCGTTTT
sh-p65-2	CCGGCACCATCAACTATGATGAGTTCTCGAGAACTCATCATAGTTGATGGTGTTTTT
sh-HADC3-1	CCGGCCTTCCACAAATACGGAAATTCTCGAGAATTTCCGTATTTGTGGAAGGTTTTT
sh-HADC3-2	CCGGCGGTCTCTATAAGAAGATGATCTCGAGATCATCTTCTTATAGAGACCGTTTTT

Table S2. Information of antibodies

Antibodies	Source	Identifier	Working dilution
Rabbit monoclonal anti-PD-L1	Cell Signaling Technology	Cat # 13684S; RRID: AB_2687655	1:1000
Rabbit polyclonal anti-Flag-tag	Proteintech	Cat # 20543-1-AP; RRID: AB11232216	1:1000
Rabbit monoclonal anti-ERK2	Cell Signaling Technology	Cat # 9108; RRID: AB_2141156	1:3000
Rabbit polyclonal anti-HDAC5	Abcam	Cat # AB55403; RRID: AB_880353	1:1000
Rabbit polyclonal anti-H3K27-ac	Abcam	Cat# AB4729; RRID: AB_2118291	1:1000
Rabbit monoclonal anti-p65	Cell Signaling Technology	Cat # 8242S; RRID: AB_10860244	1:2000
Rabbit monoclonal anti-RelB	Cell Signaling Technology	Cat# 10544; RRID: AB_2797727	1:1000
Rabbit polyclonal anti-c-Rel	Cell Signaling Technology	Cat# 4727; RRID: AB_2178843	1:1000
Rabbit polyclonal anti-p52	Cell Signaling Technology	Cat# 4882; RRID: AB_10695537	1:1000
Rabbit polyclonal anti-p50	Cell Signaling Technology	Cat# 3035; RRID: AB_330564	1:2000
Rabbit monoclonal anti-p-AKT(S473)	Cell Signaling Technology	Cat# 4060; RRID: AB_2315049	1:1000
Rabbit monoclonal anti-p-p65(S536)	Cell Signaling Technology	Cat# 3033; RRID: AB_331284	1:1000
Rabbit polyclonal anti-p-p65(S311)	Abcam	Cat# ab194926	1:1000
Rabbit acetylated lysine antibody	Cell Signaling Technology	Cat# 9441; RRID: AB_331805	1:1000
Rabbit monoclonal anti-BRD4	Cell Signaling Technology	Cat# 13440; RRID: AB_2687578	1:800
Rabbit monoclonal anti-p-STAT1	Cell Signaling Technology	Cat# 9167; RRID: AB_561284	1:1000
Rabbit monoclonal anti-β-Tubulin	Cell Signaling Technology	Cat# 2128; RRID: AB_823664	1:2000
Rabbit polyclonal anti-Histone-H3	Proteintech	Cat# 17168-1-AP; RRID: AB_2716755	1:1000
Mouse monoclonal anti-IκBα	Cell Signaling Technology	Cat# 4814; RRID: AB_390781	1:1000
Mouse monoclonal anti-IKKα	Cell Signaling Technology	Cat# 11930; RRID: AB_2687618	1:1000
Mouse monoclonal anti-IKKβ	Cell Signaling Technology	Cat# 8943; RRID: AB_11024092	1:1000
Rabbit monoclonal anti-p- IKKα/β(S176/180)	Cell Signaling Technology	Cat# 2697; RRID: AB_2079382	1:1000
Mouse anti-rabbit IgG (Conformation specific) monoclonal antibody (HRP conjugate)	Cell Signaling Technology	Cat# 5127; RRID: AB_10892860	1:4000
Goat anti-mouse polyclonal antibody	AMSBIO	Cat# BA1050-1; RRID: AB_10892412	1:4000
Rabbit monoclonal anti-CD4	Abcam	Cat# ab194926; RRID: AB_2686917	1:2000
Rabbit monoclonal anti-FOXP3	Cell Signaling Technology	Cat# 12653; RRID: AB_2797979	1:1000

Table S3. Information of chemicals

Chemicals	Source	Identifier
Ruxolitinib	Tsbiochem	T1829
JSH-23	Tsbiochem	T1930
Staurosporine	Tsbiochem	T6680
LMK235	MedChemExpress	HY-18998

Table S4. Information of recombinant DNA

Recombinant DNA	Source	Identifier
Flag-HDAC5	Addgene	Cat# 58905
pCMV4-p65	Addgene	Cat# 21966

Table S5. TCGA samples included in this study

No	Sample Id	No	Sample Id	No	Sample Id
1	TCGA-F2-6879	51	TCGA-IB-A5SO	101	TCGA-IB-AAUO
2	TCGA-HZ-7919	52	TCGA-IB-A5SP	102	TCGA-IB-AAUR
3	TCGA-HZ-7922	53	TCGA-IB-A5SQ	103	TCGA-IB-AAUS
4	TCGA-HZ-7925	54	TCGA-IB-A5SS	104	TCGA-2L-AAQE
5	TCGA-HZ-7926	55	TCGA-IB-A5ST	105	TCGA-2L-AAQI
6	TCGA-IB-7644	56	TCGA-OE-A75W	106	TCGA-2L-AAQJ
7	TCGA-IB-7646	57	TCGA-PZ-A5RE	107	TCGA-3A-A9IB
8	TCGA-IB-7647	58	TCGA-Q3-A5QY	108	TCGA-3A-A9IH
9	TCGA-IB-7649	59	TCGA-US-A774	109	TCGA-3A-A9IU
10	TCGA-IB-7651	60	TCGA-US-A779	110	TCGA-FB-AAPS
11	TCGA-IB-7652	61	TCGA-US-A77E	111	TCGA-HV-AA8X
12	TCGA-IB-7885	62	TCGA-US-A77G	112	TCGA-LB-A9Q5
13	TCGA-IB-7886	63	TCGA-HV-A7OL	113	TCGA-RB-AA9M
14	TCGA-IB-7887	64	TCGA-HZ-A77O	114	TCGA-XD-AAUL
15	TCGA-IB-7888	65	TCGA-HZ-A77P	115	TCGA-2J-AAB1
16	TCGA-IB-7889	66	TCGA-IB-A6UF	116	TCGA-2J-AAB4
17	TCGA-HZ-8001	67	TCGA-IB-A6UG	117	TCGA-2J-AAB6
18	TCGA-HZ-8002	68	TCGA-LB-A7SX	118	TCGA-2J-AAB8
19	TCGA-HZ-8003	69	TCGA-RB-A7B8	119	TCGA-2J-AAB9
20	TCGA-HZ-8005	70	TCGA-US-A776	120	TCGA-2J-AABA
21	TCGA-IB-7645	71	TCGA-HZ-A77Q	121	TCGA-2J-AABE
22	TCGA-IB-7890	72	TCGA-HZ-A8P0	122	TCGA-2J-AABF
23	TCGA-IB-7891	73	TCGA-IB-A7LX	123	TCGA-2J-AABH
24	TCGA-IB-7893	74	TCGA-IB-A7M4	124	TCGA-2J-AABI
25	TCGA-IB-7897	75	TCGA-LB-A8F3	125	TCGA-2J-AABK
26	TCGA-H6-8124	76	TCGA-S4-A8RP	126	TCGA-2J-AABO
27	TCGA-HZ-8315	77	TCGA-XN-A8T3	127	TCGA-2J-AABR
28	TCGA-HZ-8317	78	TCGA-XN-A8T5	128	TCGA-2J-AABT
29	TCGA-HZ-8519	79	TCGA-YB-A89D	129	TCGA-2J-AABU
30	TCGA-HZ-8636	80	TCGA-YY-A8LH	130	TCGA-2J-AABV
31	TCGA-HZ-8637	81	TCGA-F2-A8YN	131	TCGA-3A-A9IX
32	TCGA-IB-8126	82	TCGA-HZ-A8P1	132	TCGA-3A-A9IZ
33	TCGA-IB-8127	83	TCGA-IB-AAUM	133	TCGA-3A-A9J0
34	TCGA-F2-A44G	84	TCGA-IB-AAUP	134	TCGA-FB-AAPQ
35	TCGA-F2-A44H	85	TCGA-IB-AAUT	135	TCGA-FB-AAPU
36	TCGA-FB-A4P5	86	TCGA-IB-AAUU	136	TCGA-FB-AAPY
37	TCGA-FB-A545	87	TCGA-Q3-AA2A	137	TCGA-FB-AAPZ
38	TCGA-H6-A45N	88	TCGA-S4-A8RM	138	TCGA-FB-AAQ0
39	TCGA-HV-A5A3	89	TCGA-S4-A8RO	139	TCGA-FB-AAQ1
40	TCGA-HV-A5A4	90	TCGA-YH-A8SY	140	TCGA-FB-AAQ2
41	TCGA-HV-A5A5	91	TCGA-2L-AAQA	141	TCGA-FB-AAQ3
42	TCGA-HV-A5A6	92	TCGA-2L-AAQL	142	TCGA-FB-AAQ6
43	TCGA-HZ-A49G	93	TCGA-3A-A9I5	143	TCGA-HV-AA8V
44	TCGA-HZ-A49H	94	TCGA-3A-A9I7	144	TCGA-HZ-A9TJ
45	TCGA-HZ-A49I	95	TCGA-3A-A9I9	145	TCGA-IB-AAUQ
46	TCGA-HZ-A4BH	96	TCGA-3A-A9IC	146	TCGA-XD-AAUG
47	TCGA-HZ-A4BK	97	TCGA-3E-AAAY	147	TCGA-XD-AAUH
48	TCGA-M8-A5N4	98	TCGA-3E-AAAZ	148	TCGA-XD-AAUI
49	TCGA-FB-A5VM	99	TCGA-F2-A7TX	149	TCGA-Z5-AAPL
50	TCGA-FB-A78T	100	TCGA-IB-AAUN		

Table S6. Top20 Pathways negatively correlated with HDAC5 expression in the GSEA of TCGA pancreatic cancer dataset

NAME	SIZE	ES	NES	NOM p-val	FDR q-val
GO_REGULATION_OF_ADAPTIVE_IMMUNE_RESPONSE	10	0.69948554	2.1251383	0	0.062428206
GO_REGULATION_OF_LYMPHOCYTE_MEDIATED_IMMUNITY	9	0.69419044	2.063277	0	0.09541075
GO_REGULATION_OF_T_CELL_MEDIATED_IMMUNITY	7	0.7258479	1.9704568	0	0.21024519
GO_NUCLEOBASE_CONTAINING_COMPOUND_TRANSPORT	6	0.7282455	1.8846893	0.001324503	0.2457005
GO_ADAPTIVE_IMMUNE_RESPONSE	21	0.4854957	1.8720814	0.005561736	0.25672102
GO_T_CELL_MEDIATED_CYTOTOXICITY	6	0.71677446	1.8303968	0.006648936	0.35844636
GO_REGULATION_OF_T_CELL_MEDIATED_CYTOTOXICITY	6	0.71677446	1.7940089	0.001345895	0.47393715
GO_POSITIVE_REGULATION_OF_LEUKOCYTE_MEDIATED_CYTOTOXICITY	6	0.7019476	1.7898837	0.002673797	0.42285874
GO_POSITIVE_REGULATION_OF_CELL_KILLING	6	0.7019476	1.7879941	0.006535948	0.40208283
GO_CELL_KILLING	7	0.643951	1.7634822	0.007509387	0.42018458
GO_REGULATION_OF_LEUKOCYTE_MEDIATED_CYTOTOXICITY	7	0.643951	1.7475206	0.00630517	0.4612745
GO_ANTIGEN_PROCESSING_AND_PRESENTATION_OF_ENDOGENOUS_ANTIGEN	4	0.7819561	1.7426296	0.008733625	0.4591044
GO_REGULATION_OF_CELL_KILLING	7	0.643951	1.7359372	0.007905139	0.46324936
GO_LEUKOCYTE_MEDIATED_CYTOTOXICITY	7	0.643951	1.7247698	0.01038961	0.46616864
GO_MEMORY	5	0.7083172	1.7177576	0.012362638	0.4747816
GO_NEUROTRANSMITTER_METABOLIC_PROCESS	9	0.5843662	1.717041	0.011292347	0.45831722
GO_RESPONSE_TO_GROWTH_FACTOR	21	0.4421779	1.673725	0.01540154	0.57112193
GO_MUSCLE_CELL_DIFFERENTIATION	8	0.5912744	1.6658747	0.011378002	0.5890569
GO_NEGATIVE_REGULATION_OF_BLOOD_PRESSURE	3	0.8071216	1.6198041	0.020408163	0.7941249
GO_REGULATION_OF_CYSTEINE_TYPE_ENDOPEPTIDASE_ACTIVITY	7	0.592651	1.6138635	0.031454783	0.80549866
